# Microbial diversity in a submarine carbonate edifice from the serpentinizing hydrothermal system of the Prony Bay (New Caledonia) over a 6-year period

**DOI:** 10.3389/fmicb.2015.00857

**Published:** 2015-08-27

**Authors:** Anne Postec, Marianne Quéméneur, Méline Bes, Nan Mei, Fatma Benaïssa, Claude Payri, Bernard Pelletier, Christophe Monnin, Linda Guentas-Dombrowsky, Bernard Ollivier, Emmanuelle Gérard, Céline Pisapia, Martine Gérard, Bénédicte Ménez, Gaël Erauso

**Affiliations:** ^1^Aix-Marseille Université, Centre National de la Recherche Scientifique/INSU, Université de Toulon, IRD, Mediterranean Institute of Oceanography, UM 110Marseille, France; ^2^Institut pour la Recherche et le Développement Centre de NouméaNouméa-Nouvelle-Calédonie, France; ^3^Géosciences Environnement Toulouse, Université de Toulouse/Centre National de la Recherche Scientifique/IRDToulouse, France; ^4^Institut de Physique du Globe de Paris, Sorbonne Paris Cité, Université Paris Diderot, Centre National de la Recherche Scientifique, UMR7154Paris, France; ^5^Institut de Minéralogie et de Physique des Milieux Condensés, Université Pierre et Marie CurieParis, France

**Keywords:** hydrothermal, serpentinization, alkaline, Prony, microbial communities

## Abstract

Active carbonate chimneys from the shallow marine serpentinizing Prony Hydrothermal Field were sampled 3 times over a 6 years period at site ST09. Archaeal and bacterial communities composition was investigated using PCR-based methods (clone libraries, Denaturating Gel Gradient Electrophoresis, quantitative PCR) targeting 16S rRNA genes, methyl coenzyme M reductase A and dissimilatory sulfite reductase subunit B genes. *Methanosarcinales (Euryarchaeota)* and *Thaumarchaea* were the main archaeal members. The *Methanosarcinales*, also observed by epifluorescent microscopy and FISH, consisted of two phylotypes that were previously solely detected in two other serpentinitzing ecosystems (The Cedars and Lost City Hydrothermal Field). Surprisingly, members of the hyperthermophilic order *Thermococcales* were also found which may indicate the presence of a hot subsurface biosphere. The bacterial community mainly consisted of *Firmicutes, Chloroflexi, Alpha*-, *Gamma*-, *Beta*-, and *Delta-proteobacteria* and of the candidate division NPL-UPA2. Members of these taxa were consistently found each year and may therefore represent a stable core of the indigenous bacterial community of the PHF chimneys. *Firmicutes* isolates representing new bacterial taxa were obtained by cultivation under anaerobic conditions. Our study revealed diverse microbial communities in PHF ST09 related to methane and sulfur compounds that share common populations with other terrestrial or submarine serpentinizing ecosystems.

## Introduction

Serpentinization is the process of aqueous alteration of ultramafic rocks that releases hydrogen (H_2_), methane (CH_4_), small organic compounds, and generates alkaline fluids with pH values commonly above pH 10, at reducing conditions (McCollom, 2007; Schrenk et al., 2013). This last decade, low temperature serpentinizing environments have attracted much scientific interest because they are considered as analogs of those from which life may have arisen on the early Earth. Indeed, their particular geochemical settings may have led, a long time ago, to the transition from the abiotic reactions to biologically mediated reactions probably similar to some present day microbial metabolisms (Schulte et al., 2006; Russell et al., 2010). However, there are still only few studies of the microbiology of modern serpentinizing systems and most concerns continental sites such as the alkaline groundwater (pH 11.4) of Cabeço de Vide Aquifer (CVA, Portugal) (Tiago and Veríssimo, 2013), the ophiolitic complexes of Leka (Norway) (Daae et al., 2013), Tablelands (Newfoundland) (Brazelton et al., 2012) or The Cedars springs (California) (Suzuki et al., 2013). All these sites are readily accessible for sampling and *in situ* analyses while exploration of active serpentinization in oceanic settings is more challenging (Schrenk et al., 2013).

The Lost City Hydrothermal Field (LCHF) located at a depth of about 800 m below sea level (mbsl) on the top of the Atlantis massif at 30°N near the Mid-Atlantic Ridge is to date the most extensively studied submarine site driven by active serpentinization. There, high carbonate chimneys vent clear alkaline fluids (pH 9–11) at moderately high temperatures (40–90°C) (Kelley et al., 2001, 2005; Proskurowski et al., 2008). Molecular surveys have revealed a surprisingly low archaeal diversity inside these carbonate chimneys dominated by a single phylotype of *Methanosarcinales*, the LCMS (Lost City *MethanoSarcinales*), potentially involved in methane production or oxidation along with archaeal anaerobic methane oxidizers of the ANME-1 group (Schrenk et al., 2004; Brazelton et al., 2006, 2011). Bacterial sulfate reducers (namely *Desulfotomaculum)* were identified while microbial H_2_ production by anaerobic *Clostridiales* was evidenced by metagenomics (Brazelton et al., 2012).

**Figure 1:**
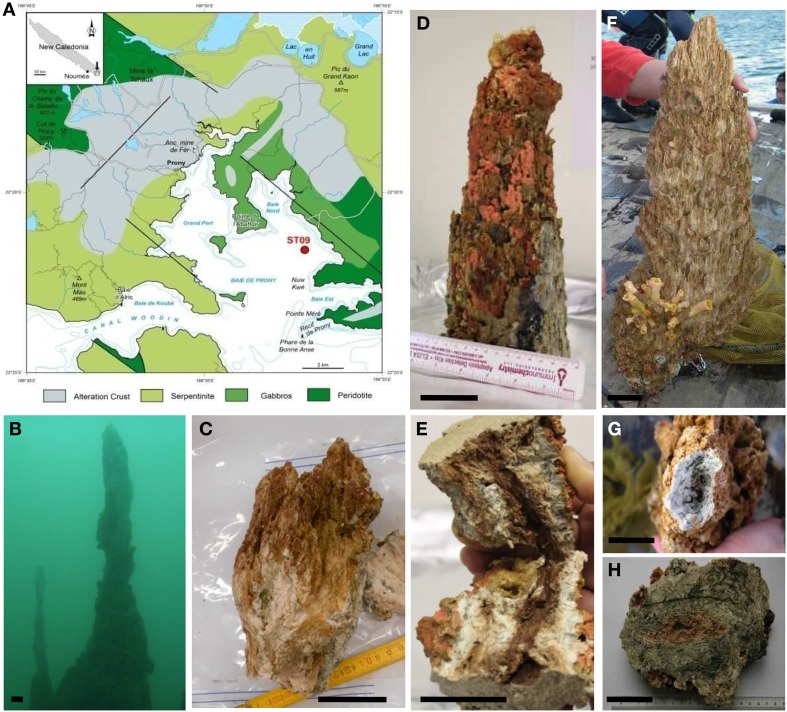
**Sampling site and chimney sample photographs**. **(A)** Map of the Bay of Prony in South New Caledonia indicating the hydrothermal sampling site ST09 located at 45–49 mbs in the East side of the Bay (after Maurizot and Vendé-Leclerc, 2009). **(B)** *In situ* photographs of the main edifice at ST09 around which chimneys were collected in 2011. **(C)** Chimney 2011 subsampling. **(D)** Top of the chimney collected in 2010 encrusted with coral ascidia and sponges. **(E)** Longitudinal fracture of the 2010 top chimney showing a mucilaginous dark-pink biofilm-like texture around the conduit. **(F)** Chimney collected in 2005 (Pelletier et al., 2006), **(G)** white top of the 2005 chimney, **(H)** radial zonation in chimney 2005 with dark gray to green intermediary parts. Scale bars: 5 cm.

Until recently, LCHF was thought to be drastically different from any other ecosystem previously discovered. However, recent geochemical and microbiological studies of the hydrothermal system of the Prony Bay (New Caledonia, SW Pacific) have revealed important similarities with the LCHF ecosystem. PHF is a marine hydrothermal system located at shallow depths in the Prony bay, on a serpentinized peridotite nappe (Figure 1A). In 2004 and 2005, bathymetric mapping and scuba dives during scientific cruises on the *R/V Alis* revealed that, besides a impressive submarine edifice called “L'Aiguille de Prony,” the floor of the Prony Bay hosted several 2–10 m high domes and pinnacles at water depths between 30 and 50 m (Pelletier et al., 2006). A recent geochemical study has shown that the hydrothermal hyperalkaline (pH 11) end member has a meteoric origin (run off waters) and is enriched in H_2_ and CH_4_ (Monnin et al., 2014). The first study of the microbial community of PHF showed that archaeal diversity was low and dominated by *Methanosarcinales* while members of *Chloroflexi, Firmicutes*, and *Proteobacteria* phyla were the most abundant bacteria. It also showed variation in microbial community structure, abundance, and diversity depending on the location of the sampling site in the Prony Bay, probably related to change in environmental factors such as water depth and maturity (age) of the carbonate chimneys (Quéméneur et al., 2014).

Very few culture-dependent approaches have been reported to characterize the bacterial populations from serpentinite environments and are so far restricted to alkaline groundwaters of Oman, CVA and the Cedars (Bath et al., 1987; Schrenk et al., 2013; Tiago and Veríssimo, 2013; Suzuki et al., 2014). In CVA, only the aerobic heterotrophic fraction of the cultivable microbial community was targeted and was found to be mainly constituted of Gram-positive bacteria belonging to *Actinobacteria, Bacillus* and *Staphylococcus* (Tiago et al., 2004). Recently, aerobic hydrogen-oxidizers growing optimally at pH 11 were isolated from the continental serpentinizing springs at The Cedars and proposed as representing a new genus, “*Serpentinomonas*,” within the *Betaproteobacteria* (Suzuki et al., 2014).

Here, we describe the microbial communities inhabiting three active PHF chimneys collected over a 6-year period from one of the deepest hydrothermal sites not included in the previous study (Quéméneur et al., 2014) using a combination of PCR-based methods (DGGE, qPCR and sequencing of clone libraries of 16S rRNA gene and functional genes), microscopy (CSLM and FISH) and anaerobic cultivation methods.

## Materials and methods

### Sampling site and sample collection

The sampling site (22°21.653S, 166°52.777E, site ST09 on Figure 1A) is an active hydrothermal area located East of the Central Bay of Prony, in the south of New Caledonia. The general geological context and the composition of venting fluids and gasses are given by Monnin et al. (2014) (Supplementary Table 1).

The upper parts of three active chimneys, venting alkaline fluids, were collected by SCUBA diving on the western part of a hydrothermal carbonated edifice with a dome shape based in a depression at a water depth of about 50 m. The first chimney collected in June 2005 during the first exploratory campaign on *Alis* RV (Pelletier et al., 2006) at 49 mbsl was 62 cm high with 22 cm maximal diameter; the second one collected in November 2010 at 47 mbsl was about 30 cm high and 14 cm maximal diameter and the third one collected in November 2011 during the HYDROPRONY campaign on *Alis* RV (Monnin et al., 2014) at 45 mbsl was 22 cm height with 15 cm maximal diameter (Figures 1B–H). The three sampled chimneys possessed very fragile crumbly white needles at their summit (Figure 1G), testifying to active fluid discharge. They were more or less encrusted with various invertebrates such as ascidia, coral and marine sponges (Figure 1D) attesting for their long-lasting existence and advanced stage. They were mainly composed of aragonite, brucite, calcite, and magnesium carbonates as detailed elsewhere (Pisapia et al., 2013). The chimneys collected in 2005 and 2011 were recovered aboard the *Alis* RV. In 2005, the chimney was quickly frozen aboard at −20°C, sent to the IRD research center in Nouméa, sawed in still frozen slices about 5 cm thick and stored at −80°C. The chimmey collected in 2011 was directly sawed in sections and subsampled aboard the *Alis* RV, for molecular biology and culture analyses. In 2010, the chimney recovered aboard an outboard boat was kept on ice until back (within 4–5 h) at the IRD in Nouméa where it was sawed and subsampled as described below. Cross-sections of active chimneys showed a light beige highly porous material with sometimes friable beige to pink mucilaginous material visible around the central conduit, suggesting the presence of a bacterial biofilm (Figures 1E,H). Subsamples corresponding to internal parts of the chimneys were carefully collected trying to avoid contamination by the outer parts as much as possible and stored in sterile 50 mL Falcon tubes at −80°C for later DNA extraction. For fluorescent *in situ* hybridization (FISH) experiments, samples were fixed for 3 h at 4°C in a solution of phosphate-buffered saline pH 7.2 (PBS; 130 mM NaCl, 7 mM Na_2_HPO_4_, 30 mM NaH_2_PO_4_) containing 2% (vol/vol) formaldehyde (methanol free, Ultra Pure; Polysciences), washed twice with PBS and resuspended in sterile Falcon tubes with 50% ethanol/PBS. Samples fixed in this buffer were then kept at −20°C.

Samples used for anaerobic cultivation experiments were ground aseptically with a pestle in a mortar under a continuous flux of N_2_ gas to preserve anaerobiosis and distributed into sterile glass vials which were then tightly closed with butyl rubber stoppers and crimped with aluminum seals. The headspace in the vials was filled with 100% N_2_ with a slight overpressure (100 kPa) to prevent oxygen entrance. They were stored at 4°C until use.

### DNA extraction

Crushed chimney samples (0.3 g) were suspended in 1 mL TE-Na-1X lysis buffer (100 mM Tris, 50 mM EDTA, 100 mM NaCl, 1% SDS (w/v), 1% (w/v) Sarkosyl, pH 8.0). Cells were disrupted by bead beating using Lysing Matrix E (MP, Biomedicals). After digestion with proteinase K (1 mg.mL^−1^ final) for 1 h at 55°C, DNA was extracted twice with a volume of phenol/chloroform/isoamyl alcohol (25/24/1; pH 8.0), then with a volume of chloroform. Aqueous and organic phases were separated by centrifugation at 13,000 g for 15 min at 4°C. DNA was precipitated with 0.7 volume of isopropanol, pelleted by centrifugation at maximum speed for 15 min, then air-dried and finally resuspended in 30 μL of TE 1X (10 mM Tris,.HCl, 2 mM EDTA, pH 8.0) buffer. The concentration of DNA extracts was measured using the Qubit® fluorometer (Invitrogen).

### PCR-amplification of 16s rRNA and functional genes

PCR set-up was carried out under aseptic conditions using autoclaved and/or UV-treated plasticware and pipettes. The PCR mixtures contained (total 50 μL) 0.4 pmol.μL^−1^ of primers, 1 μL of chimney DNA template (1/10 dilution), 1X reaction buffer (GoTaq® Hot Start Green Master Mix, Promega). The primer sets used for 16S rRNA gene amplification (Supplementary Table 2) were (i) for cloning purposes: 27F/907R for *Bacteria* and 109F/958R for *Archaea*, (ii) for DGGE purpose: 341F-GC/907R for *Bacteria* and 344F-GC/958R for *Archaea*. PCR reactions were carried out using a T100 thermal cycler (BioRad) with the following conditions: initial denaturation at 95°C for 2 min followed by 30 cycles, each cycle consisting of denaturation at 94°C for 30 s, primers annealing at 50°C for 30 s and extension at 72°C for 90 s, followed by a final extension step of 5 min at 72°C. The *mcrA* and *dsrB* genes were amplified as described above using the primer sets MLF/MLR and DSRp2060F/DSR4R respectively, except that 35 cycles were performed and primer hybridization was carried out at 55°C.

### Cloning and sequencing

Each gene library (archaeal and bacterial 16S rRNA, *mcrA*, and *dsrB*) was constructed from three to five independent PCR products that were pooled and purified using a Qiaquick PCR purification kit (Qiagen) before ligation in pGEM-T Easy vector (Promega) overnight at 4°C. *Escherichia coli* JM109 competent cells (Promega) were used for transformation by heat-shock using a standard protocol (Promega kit manual). White colonies were used to inoculate 96-wells microplates filled with LB plus ampicillin (100 μg.mL^−1^) agar. Plasmid Miniprep and Sanger sequencing were operated by GATC Biotech AG, Germany.

### Phylogenetic and diversity analyses of chimney communities

Partial 16S rRNA gene sequences (average size around 900 bp) were checked with the online Bellerophon program version 3 at Greengenes website (http://greengenes.lbl.gov/) to exclude the chimeric artifacts. Each sequence was compared to 16S rRNA gene sequences of the NCBI GenBank nr database by using BLAST (http://www.ncbi.nlm.nih.gov/BLAST/) (Altschul et al., 1990). 16S rRNA sequences were also phylogenetically classified according to the Naive Bayesian rRNA Classifier (Version 1.0) of the Ribosomal Database Project II (http://rdp.cme.msu.edu/). Non-chimeric 16S rRNA gene sequences and translated *dsrB* and *mcrA* sequences were aligned using Muscle (Edgar, 2004) implemented in the MEGA6 software (Tamura et al., 2013). Alignments were edited manually and regions of ambiguous alignment removed. The MOTHUR program (Schloss et al., 2009) was used to group sequences into operational taxonomic units (OTUs) with a threshold of 97% sequences identity, commonly accepted as the minimum similarity level within a prokaryote species (Gevers et al., 2005). The input files for MOTHUR were distance matrices generated by MEGA6 using a Maximum Composite Likelihood model for 16S rRNA genes. The coverage (C) of each clone library was calculated according to the equation: *C* = 1–(n/N) where n is the number of OTU and N is the total number of clones in the library (Good, 1953). Coleman rarefaction curves, Chao1 values and Shannon indices were obtained from MOTHUR. At least one sequence representing each OTU, called phylotype, here was further aligned using Muscle with related sequences retrieved from NCBI databases using BLAST tools. The evolutionary history was inferred using both the Neighbor-Joining method with the Maximum Composite Likelihood model (Saitou and Nei, 1987; Tamura et al., 2004) and the Maximum Likelihood method based on the General Time Reversible model (Nei and Kumar, 2000) using MEGA6 software. The evolutionary distances were computed using the Kimura 2-parameters method (Kimura, 1980) for *dsrB* and *mcrA* translated sequences. The data were bootstrapped 1000 times to assess support for nodes. All positions containing gaps and missing data were eliminated.

### Quantitative PCR

Total bacterial and archaeal 16S rRNA genes were quantified using the primers sets 341F/518R and 344F/519R, respectively. The primer sets used for quantifying *mcrA* and *dsrB* genes were ME3MF:ME3MF-e (250:1)/ME2r′ and DSRp2060F/DSR4R, respectively (see list of primers in Supplementary Table 2). The detailed procedure was described previously (Quéméneur et al., 2014).

### Epifluorescence microscopy, FISH and CSLM

Direct microscopic observations were carried out on the sub-samples kept anaerobically at 4°C using a phase-contrast Nikon Eclipse 6500 epifluorescence microscope with an oil-immersion objective Nikon Plan Fluor 100X, by simply crushing a drop of chimney slurry between slide and cover slides. The blue autofluorescence of methanogen cells due to their co-factor F420 content was recorded after excitation at 420 nm. Fluorescence *in situ* hybridization was carried out at 46°C for 2–4 h using 5 ng·μL^−1^ of probe in 0.9 M NaCl, 20 mM Tris-HCl pH 8, 0.01% SDS containing 35% (vol/vol) formamide. As experiments were targeting *Euryarchaeota*, the probe used was the FITC-labeled S-K-Eury-0498-a-A-14 (EURY498) (Supplementary Table 2). Samples were then washed for 15 min at 48°C in 20 mM Tris pH 8, 5 mM EDTA, 0.01% SDS, 0.084 mM NaCl, soaked in cold water for a few seconds and air dried. Samples were then stained with DAPI or Syto®9, (blue and green fluorescent nucleic acid stains at concentration of 1 μg·mL^−1^ (Invitrogen) for 1 min, then washed for a few seconds in cold water and left to dry. Hybridized cells, covered by Citifluor AF3 (glycerol mountant solution from Emgrid), were examined at the Institut de Physique du Globe de Paris using an Olympus FluoView FV1000 confocal laser scanning microscope with a spectral resolution of 2 nm and a spatial resolution of 0.2 mm. The microscope is equipped with a 405-nm laser diode and multiline argon (458, 488, and 515 nm), helium–neon–green (543 nm) and helium–neon–red (633 nm) lasers. Observations were carried out with an oil immersion UPLSAPO 60XO objective (Olympus; x60 magnification, numerical aperture = 1.35). Fluorescence image stacks were obtained with concomitant excitation at wavelengths of 405 and 488 nm by collecting the emitted fluorescence between 425–475 and 500–530 nm, respectively. The fluorescence spectra of the F_420_ cofactor, implied in the energy metabolism of methanogens (Doddema and Vogels, 1978) were recorded between 405 and 800 nm for a wavelength excitation of 405 nm, with band width analyses of 10 nm and steps of 5 nm. Three-dimensional images were acquired, visualized and processed using the F10-ASW FLUOVIEW software (Olympus).

### Denaturing gradient gel electrophoresis (DGGE)

Total DNA of the 2005, 2010, and 2011 chimney samples were used as templates for the PCR amplification of the 16S rRNA gene V3 segments, which were profiled using DGGE. Aliquots of 25 μL of PCR products per lane were loaded on gels using a D-Code universal mutation detection apparatus (Bio-Rad, CA, USA). Electrophoresis was conducted in 6% (w/v) acrylamide gel with 40–70% denaturing gradient in 1 × TAE buffer (20 mM Tris acetate, 10 mM sodium acetate, and 0.5 mM EDTA) at a constant voltage of 200 V at 60°C for approximately 5 h. The gel was stained with SybRGold, de-stained with deionized water, and finally scanned using the Gel Doc image acquisition system (Bio-Rad, CA, USA). The predominant DGGE bands were then excised from the gels, eluted overnight in 50 μL sterile deionized water, and used as templates to re-amplify the 16S rRNA gene-V3 segments by using the same PCR primer pairs but without the GC clamp. The PCR products were then sequenced at GATC Biotech AG, Germany. Taxonomic affiliation of the PCR product sequence was based on the results of BLASTN against the nr database at NCBI. Local BLASTN was also used to compare these DGGE sequences (~600 pb) with our database containing all 16S rRNA sequences from both archaeal and bacterial clone libraries from PHF.

### Estimating the growth temperature of archaea

The method is based on the strong correlation between the growth temperatures of cultured prokaryotes and the guanine-plus-cytosine contents (P_GC_) of their 16S rRNA sequences, thermophilic and hyperthermophilic strains having higher P_GC_ than those of psychrophilic and mesophilic isolates (Galtier et al., 1999; Khachane et al., 2005; Kimura et al., 2006, 2007; Wang et al., 2006). Kimura et al. (2010, 2013) proposed linear regression equations to infer minimum (T_min_), optimum (T_opt_) and maximum (T_max_) growth temperatures of not-yet cultured prokaryotes and of microbial communities based on the P_GC_ of their 16S rRNA sequences and they showed that these estimates correlate well with the *in situ* temperatures (Kimura et al., 2010, 2013). We therefore used their equations to estimate the growth temperatures of the main archaeal phylotypes encountered in the PHF chimneys. These calculations were possible since the sequences of our clones encompassed the same internal region of the 16S rRNA as that used by Kimura et al. (2013), which is between the *Archaea*-specific primers 109F and 915R. All the 16S rRNA sequences of our clones, of reference strains and from other studies, were aligned using MEGA 6 and manually trimmed to be of the same length (800 bp) as those used in Kimura et al. (2013) linear regressions. Their GC content was calculated on line at http://www.endmemo.com/bio/gc.php and their respective T_min_, T_opt_, and T_max_ were calculated using Kimura's equations in a spreadsheet.

### Anaerobic cultivation experiments

Various culture media were designed targeting enrichment of: (i) methanogens, using either acetate, formate, methanol, mono, and tri-methylamine (MMA and TMA), all at 20 mM, or H_2_/CO_2_ (80/20, 200 kPa) as substrates for methanogenesis, (ii) sulfate and sulfur-reducers using either yeast extract (5 g·L^−1^), glucose (5 g·L^−1^), acetate (20 mM), lactate (20 mM) or H_2_/CO_2_(80/20, 200 kPa) as electron donors with either sulfate, thiosulfate (both 20 mM) or elemental sulfur (3 g·L^−1^) as electron acceptors. The basal medium (BM) used for these cultures contained (g·L^−1^): NH_4_Cl (1.0), K_2_HPO_4_ (0.3), KH_2_PO_4_ (0.3), KCl (0.1), CaCl_2_·2H_2_O (0.1), NaCl (2, 10, 20, or 30), yeast extract (Difco) (0.1), cysteine hydrochloride (0.5) and 1 mL trace mineral element solution (Widdel et al., 1983). Media were boiled and distributed under strict anaerobic conditions in Hungate tubes or penicillin vials, then sterilized by autoclaving. Prior to inoculation, the pH was adjusted to 9 to 10.5, with a solution of 8% (w/v) Na_2_CO_3_. Hungate tubes (5 mL medium) were then supplemented with 0.1 mL of 3% (w/v) Na_2_S·9H_2_O, 0.1 mL of 15% (w/v) MgCl_2_·6H_2_O and aliquots of stock solutions of the substrates (20 mM, otherwise 5 g·L^−1^ for complex organic matter). Enrichment cultures were inoculated with about 5% (v/v) of anaerobic chimney slurry and incubated at 30, 37, 50, and 60°C. They were regularly checked for growth by visual observation of fresh aliquots with a light microscope or by measurement of the gas production (H_2_, CO_2_, CH_4_, or H_2_S) by gas chromatography as described previously (Mei et al., 2014). Strains were isolated by picking up colonies formed on 1.6% agar solid medium using Hungate's roll tubes method (Hungate, 1969). They were deemed pure after repeating the roll tubes step at least twice and finally transferred into liquid medium for stock cultures.

### Nucleotide sequence accession numbers

All the sequences obtained in this study were deposited at Genbank under accession number from KJ149101 to KJ149254 and KJ159188 to KJ159206.

## Results and discussion

### Microbial community abundance, structure and variation over the 6 year period

Real-time quantitative PCR was used to assess the relative abundance of archaea and bacteria within three PHF chimneys. The abundance of bacterial 16S rRNA genes was more than one order of magnitude larger than that of archaeal 16S rRNA genes each year (Table 1). The bacterial 16S rRNA gene abundance ranged from 4.0 ± 0.9 × 10^7^ (2010) to 9.1 ± 0.1 × 10^7^ (2005) whereas the archaeal 16S rRNA gene abundance ranged from 3.3 ±0.8 × 10^6^ (2005) to 4.5 ± 0.9 × 10^6^ (2010) copies per g of wet weight (gww). No significant difference was observed between the sampling years (*P*>0.05). Archaea accounted for 9.8 (2005) to 25.3% (2010) of the total prokaryotic cells, given an average 16S rDNA copy number in archaeal and bacterial genomes of 1.2 and 3.6, respectively (Fogel et al., 1999; Klappenbach et al., 2001). At LCHF, in comparison, the total number of DAPI stained cells counted by epifluorescence microscopy in the active venting chimneys varied from 0.02 to 8.4 × 10^8^ cells per gww, with archaea representing between 55 and 77% of the total microbial cells as determined by FISH experiments (Kelley et al., 2005). The *dsrB* gene abundance ranged from 3.4 ± 0.6 × 10^5^ (2010) to 1.1 ± 0.4 × 10^6^ (2005) copies per gww, while the *mcrA* abundance varied from 1.0 ± 0.1 × 10^6^ (2005) to 1.8 ± 0.3 × 10^6^ (2011) copies per gww. Assuming that archaeal and bacterial cells respectively contain only one copy of the *mcrA* or *dsrB* gene (Klein et al., 2001; Luton et al., 2002), the methanoarchaea accounted for 35.6% (2010) to 60.2% (2011) of the archaea, whereas the percentage of sulfate-reducers was less than 6% of the bacteria. While such quantitative PCR data are not available for other hyperalkaline systems, LCHF FISH experiments showed *Methanosarcinales* specific probe hybridized between 68 and 80% of the archaeal cells.

**Table 1:** **Gene abundance and cell number estimates from quantitative PCR on bacterial and archaeal 16S rRNA genes and on ***dsrB*** and ***mcrA*** genes and diversity indices for their respective clone libraries**.

**Chimney sampling year**	**Library**	**Number of gene copies per g (ww) of chimney**	**Number of cells estimated per g (ww) of chimney**	**OTUs^a^/Clones**	**Coverage**	**Chao1^b^**	**Shannon^b^**
2005	16S *Bacteria*	9.1 ± 0.1 × 10^7^	2.5 ± 0.1 × 10^7^	15/71	0.79	18.75 (15.63–37.02)	2.20 (2.47–1.97)
	16S *Archaea*	3.3 ± 0.8 × 10^6^	2.7 ± 0.8 × 10^6^	3/96	0.97	3 (3-ND)	0.48 (0.33–0.63)
	*dsrB*	1.1 ± 0.4 × 10^6^	1.1 ± 0.4 × 10^6^	6/28	0.79	5 (4.07-17.27)	0.92 (0.66–1.18)
	*mcrA*	1.0 ± 0.1 × 10^6^	1.0 ± 0.1 × 10^6^	2/32	0.94	2 (2-2)	0.64 (0.52–0.76)
2010	16S *Bacteria*	4.0 ± 0.9 × 10^7^	1.1 ± 0.9 × 10^7^	55/109	0.54	385 (173.12-976.88)	3.46 (3.23–3.70)
	16S *Archaea*	4.5 ± 0.9 × 10^6^	3.7 ± 0.9 × 10^6^	21/119	0.82	49 (28.41-126.69)	2.45 (2.26–2.64)
	*dsrB*	3.4 ± 0.6 × 10^5^	3.4 ± 0.6 × 10^5^	1/29	0.97	1 (1–1)	0 (–0.047–0.047)
	*mcrA*	1.3 ± 0.1 × 10^6^	1.3 ± 0.1 × 10^6^	1/27	0.96	1 (1–1)	0 (–0.081–0.081)
2011	16S *Bacteria*	6.7 ± 0.1 × 10^7^	1.9 ± 0.1 × 10^7^	33/80	0.64	64.66 (43.39–129.45)	3.03 (2.80–3.27)
	16S *Archaea*	3.5 ± 0.6 × 10^6^	2.9 ± 0.6 × 10^6^	14/90	0.84	21.5 (15.32–56.53)	1.65 (1.37–1.93)
	*dsrB*	1.0 ± 0.2 × 10^6^	1.0 ± 0.2 × 10^6^	6/33	0.82	6 (6-ND)	1.29 (0.99–1.58)
	*mcrA*	1.8 ± 0.3 × 10^6^	1.8 ± 0.3 × 10^6^	4/29	0.86	5 (4.07–17.27)	0.92 (0.66–1.19)

a OTUs (operational taxonomic units) defined at 3% sequence differences (nucleotide or amino acids) threshold.

b Ranges of diversity indices include 95% confidence intervals as calculated by MOTHUR (Schloss et al., 2009). WW, Wet Weight; ND, Not Determined.

DGGE profiling of the bacterial and archaeal communities in the ST09 chimneys was performed for each sampling year. Bacterial profiles were very similar although for the 2010 sample, the major bands were less visible and included an additional band (n°5) (Supplementary Figure 1). The corresponding sequences were affiliated to *Firmicutes, Chloroflexi*, and *Bacteroidetes* (Table 2). Archaea profiles were also quite similar for 2005 and 2011 samples contrasting with the 2010 sample which contained only one distinct band (n°11) particularly intense, affiliated to the *Thaumarchaeota* (*Cenarchaeales*). All other archaea detected using DGGE belonged to the *Methanosarcinales.* Most of them (bands n° 7, 8, 9, 10, and 12) shared 97–99% sequence identity with the Ced_A01 clone from The Cedars springs whilst one band (n°6) was affiliated to the LCMS cluster.

**Table 2:** **Phylogenetic affiliations of the 16S rRNA sequences obtained from PCR-DGGE analyzes**.

**DGGE band N°**	**Phylogenetic affiliation**	**Closest PHF phylotype (BLAST identity %)**	**Closest environmental sequence (BLAST identity %)**	**Origin**
DG-Bac1-341F (KJ149114)	*Firmicutes*	PHF_13-B21_J06 (KJ149248) (99%)	clone B11-15_GoMY (AB476673) (98%)	Sulfur containing freshwater source
DG-Bac2-341F (KJ149115)	*Chloroflexi*	PHFST12_B1 (KF886112) (98%)	clone KM35B-155 (AB300104) (96%)	Holocene mud sediment
DG-Bac3-341F (KJ149116)	*Firmicutes*	PHF_15-B29_J20 (KJ149238) (92%)	clone ML635J-16 (AF507885) (91%)	Mono lake at a depth of 35 m
DG-Bac4-341F (KJ149117)	*Firmicutes*	PHF_15-B34_D22 (KJ159205) (100%)	clone 56S_1B_61 (DQ837275) (95%)	Pristine coastal aquifer, Spain
DG-Bac5-341F (KJ149118)	*Bacteroidetes*	PHF-2HY3BaC08 (KJ149173) (83%)	clone OTU199_Ref_Clone01 (AB694464) (95%)	Deep-sea sediment
DG-Arc6-Saf (KJ149119)	*Methanosarcinales*	PHF_2AarcF09 (KJ149145) (100%)	clone SGXU600 (FJ791432) (98%)	Carbonate chimney in Lost City
DG-Arc7-Saf (KJ149120)	*Methanosarcinales*	PHF_13-A45_G13 (KJ149156) (97%)	clone Ced_A01 (KC574884) (97%)	Serpentinizing springs in The Cedars
DG-Arc8-Saf (KJ149121)	*Methanosarcinales*	PHFST07_A2 (KF886032) (99%)	clone Ced_A01 (KC574884) (99%)	Serpentinizing springs in The Cedars
DG-Arc9-Saf (KJ149122)	*Methanosarcinales*	PHF_13-A45_G13 (KJ149156) (98%)	clone Ced_A01 (KC574884) (98%)	Serpentinizing springs in The Cedars
DG-Arc10-Saf (KJ149123)	*Methanosarcinales*	PHF_13-A45_G13 (KJ149156) (99%)	clone Ced_A01 (KC574884) (98%)	Serpentinizing springs in The Cedars
DG-Arc11-Saf (KJ149124)	*Thaumarchaeota*	PHF_13-A02_M23 (KJ149153) (100%)	clone SPCiA-7 (KF036083) (99%)	Marine sponge, *Cinachyra* sp.
DG-Arc12-Saf (KJ149125)	*Methanosarcinales*	PHF_13-A45_G13 (KJ149156) (99%)	clone Ced_A01 (KC574884) (99%)	Serpentinizing springs in The Cedars

The diversity of the microbial community was assessed for each sample by clone library sequences of 16S rRNA and functional genes (*dsrB, mcrA*). The number of OTUs, the values of the richness estimators (Table 1) and the shape of the rarefaction curves (Supplementary Figure 2) obtained from 16S rRNA gene sequence analyses confirmed that archaea were much less diverse than bacteria in each sample. The coverage was the highest for the 2005 clone libraries and the lowest for the 2010 clone libraries, even if the latter counted a higher number of clones, reflecting a higher diversity in 2010 samples than in 2005.

Archaeal diversity was well covered with values ranging from 79 to 97% (Table 1), suggesting that dominant members of the archaeal community were represented in the clone libraries, as observed in recent studies on microbial communities from serpentinization-driven subterrestrial ecosystems (Daae et al., 2013; Suzuki et al., 2013). Archaeal 16S rRNA sequences (*n* = 305) belonged to the phyla *Euryarchaeota* (69%) or *Thaumarchaeota* (31%).

**Figure 2:**
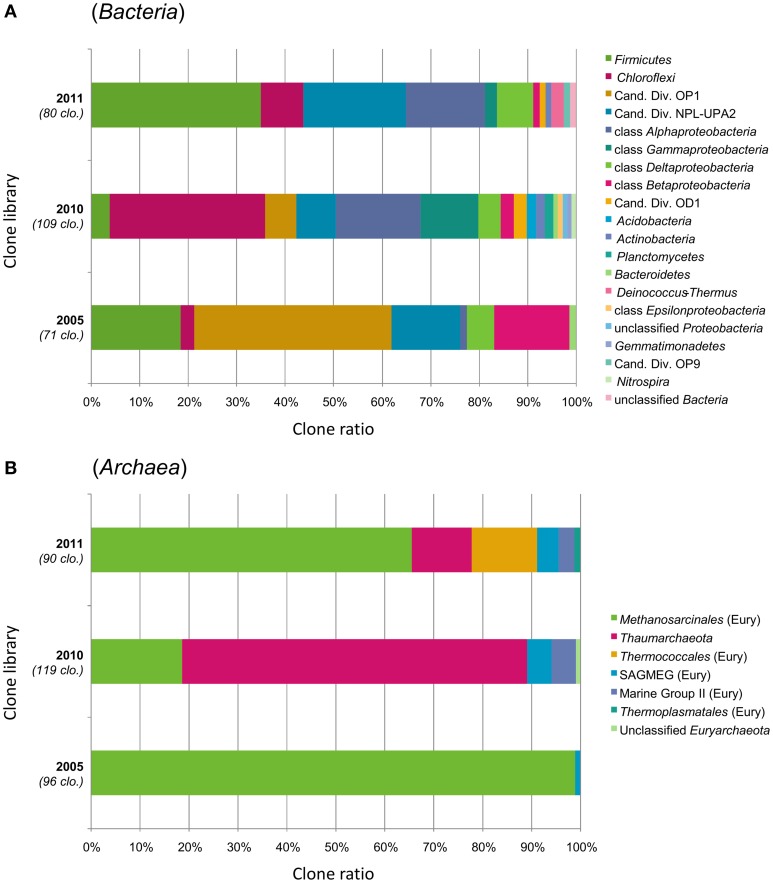
**Prokaryotic community structure in the interior parts of ST09 Prony chimneys based on phylum-level taxonomic distribution of the (A) bacterial and (B) archaeal 16S rRNA gene clones**. Phylum *Proteobacteria* is divided into class-level taxons. Sampling years are shown on the left of the bar charts (2005, 2010, and 2011) with the number of analyzed clones. “Cand. Div.” stands for Candidate Division; SAGMEG, South Africa Gold Mine Euryarchaeotic Group; “Eury,” Euryarchaeota.

Coverage values of the bacterial clone libraries ranged from 54% (2010) to 79% (2005), indicating that more clones may be needed for an adequate estimation of the bacterial diversity. Even so, we considered that the major bacterial groups were represented. Bacterial 16S rRNA gene sequences (*n* = 260) spanned many phyla (Figure 2). In average (over the three chimneys sampled), the *Firmicutes* phylum was the most represented (17.3% of total bacterial clones), followed by the *Chloroflexi* (16.9%), Candidate Divisions OP1 (13.8%), and NPL-UPA2 (13.8%), *Alphaproteobacteria* (12.7%), *Betaproteobacteria* (5.8%)*, Gammaproteobacteria* (5.8%) and *Deltaproteobacteria* (5.8%). To a lesser extent (≤1.5% of bacterial clones), Candidate Division OD1, *Acidobacteria, Actinobacteria, Planctomycetes, Bacteroidetes, Deinococcus-Thermus, Epsilonproteobacteria, Gemmatimonadetes*, candidate division OP9 and *Nitrospira* were also detected.

The predominance of *Euryarchaeota (Methanosarcinales), Firmicutes*, and *Chloroflexi* was also attested by DGGE. Both clone libraries composition and DGGE profiles suggest that the microbial communities were similar in 2005 and 2011, while *Thaumarchaeota, Gamma*-, and *Betaproteobacteria* corresponding to putative aerobic microorganisms were more abundant in 2010. The changes in population structure observed through the years may be explained by temporal variation in hydrothermal activity of the site in terms of discharge rate and composition of the fluid (Brazelton et al., 2006, 2010a). Unfortunately, we do not have such time series for ST09 fluid geochemistry because fluid was not sampled in 2005 and because of the difficulties to collect undiluted fluid samples by SCUBA diving (especially in deep sites). We obtained reliable chemical composition of the fluid in ST09 only since 2011 (Monnin et al., 2014). Distinct chimneys were sampled in 2005, 2010, and 2011. Although, they seemed to be at a roughly similar stage of formation, heterogeneity in the mineralogy or porosity of the subsamples as well as variation in the colonization of their outer wall by invertebrates may have influenced the permeability of the carbonate concretion and therefore the level of mixing with the surrounding seawater which can explain the marked difference of the microbial distribution in the chimney collected in 2010. Another possible explanation could lie in the fact that the 2010 chimney could not be processed as quickly as the two others and therefore that aerobic microorganisms from the outer parts of the chimney exposed to seawater may have diffused in its interior.

### Two dominant *Methanosarcinales* clusters (LCMS and TCMS) coexist at PHF

**Figure 3:**
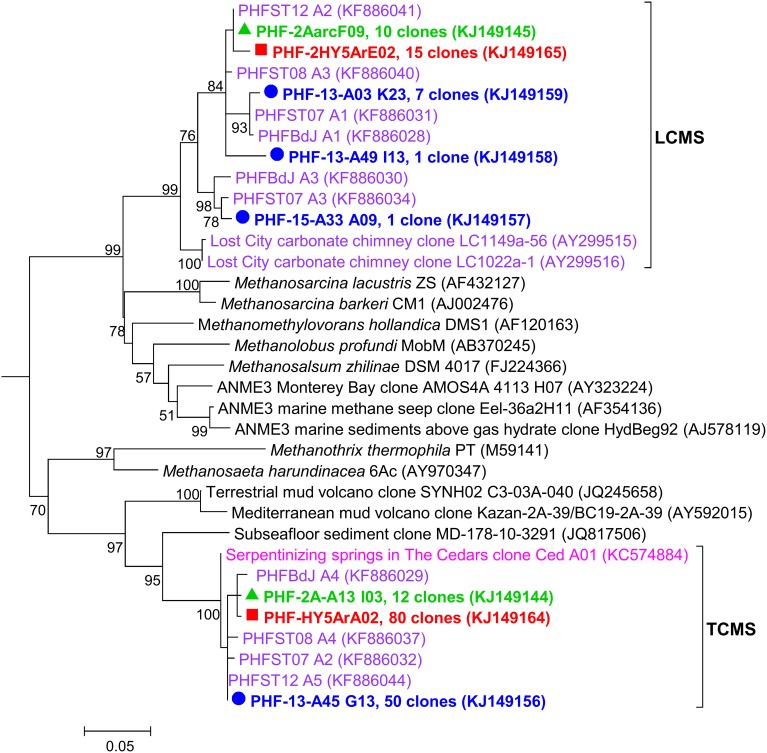
**Maximum likelihood phylogenetic tree representing *Methanosarcinales* (among *Euryarchaeota*) 16S rRNA gene phylotypes from ST09 chimneys**. Based on 489 aligned positions. The tree was constructed using the Maximum likelihood method. Bootstrap values < 50% are not shown. Clone libraries are distinguished by various symbols and colors: 2005 (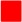 in red), 2010 (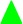 in green), and 2011 (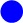 in blue). Purple font refers to phylotypes from marine hydrothermal serpentinized systems, pink font refers to phylotypes from other terrestrial alkaline sites. Scale bar, number of substitutions per site.

One of the most salient results from the 16S rRNA diversity survey was the presence of two major clusters belonging to the *Methanosarcinales* order that largely dominated the whole archaeal population (up to 99% of the clones in 2005). Both clusters were detected in 2005, 2010, and 2011 samples (Figure 3) and their predominance was supported by DGGE results (Table 2, Supplementary Figure 1).

Three PHF phylotypes (PHF-2A-A13 I03, PHF-HY5ArA02, and PHF-13-A45 G13) totaling 142 clones closely matched with the unique (and minor) archaeal phylotype Ced-A01 detected so far only in The Cedars terrestrial serpentinizing springs (here named TCMS by analogy with the LCMS of Lost City), while the closest cultivated organisms were of the *Methanosaeta* genus (=86% identity) known as obligate acetoclastic methanogens (Suzuki et al., 2013). The second cluster contained five phylotypes (PHF-2AarcF09, PHF-2HY5ArE02, PHF-13-A03 K23, PHF-13-A49 I13, and PHF-15-A33 A09) totaling 34 clones that were clearly affiliated to the famous LCMS described at Lost City (93.4–95.8% identity with the LCMS clones LC1022a-1 and LC1149a-56) (Figure 3). Previous studies showed that LCMS cells represented up to 80% of the total cells in biofilms coating active carbonate chimneys of LCHF (Brazelton et al., 2010a,b). Accordingly, the seven *mcrA* OTUs obtained (over a total of 88 clones) fall into a single group within the *Methanosarcinales* order suggesting that all the methanoarchaea of PHF ST09 chimneys belonged to this order (Supplementary Figure 3). They were all closely affiliated with uncultured LCHF *mcrA* sequences (90.0–91.8% identity) (Kelley et al., 2005) and more distantly related to *mcrA* group f sequences of ANME-3 (75.6–83.7% identities) (Lösekann et al., 2007) (Supplementary Figure 3). There was no sequence of *mcrA* reported for TCMS (Suzuki et al., 2013) as reference, therefore, it was difficult to foresee where such sequences will branch in a *mcrA* phylogenetic tree. However, at the closest similarity level, two distinct clusters can be distinguished within PHF *mcrA* sequences, the first one (15-M25, 15-M26, and 15-M1) contains 16% of the total clones (all years) while the second (2-C-M1, 23-M22, 23-M1, 15-M3) represents 84% of the total clones, a ratio that roughly coincides with the ratio of LCMS-like vs. TCMS-like ratio of the 16S rRNA gene sequences, 19 vs. 81% respectively.

These two dominant *Methanosarcinales* phylotypes were up to now considered as unique and specific to two highly contrasted serpentinizing ecosystems, one fully submarine (LCHF type) and the other fully continental (The Cedars). Their coexistence in PHF chimneys is remarkable and may reflect the singularity of PHF representing a transition between these two systems: the discharge of hydrothermal alkaline freshwater of continental origin in a shallow marine environment (Monnin et al., 2014). We propose that this *Methanosarcinales* group represents a hallmark of serpentinization-hosted ecosystems. The surprising higher abundance of TCMS over LCMS may be due to the low salinity of PHF fluids of meteoric origin, such as it is at The Cedars, but different from the fully marine LCHF. Thus, depending on the mixing degree of the low salinity fluid with surrounding seawater, both LCMS and TCMS may coexist.

**Figure 4:**
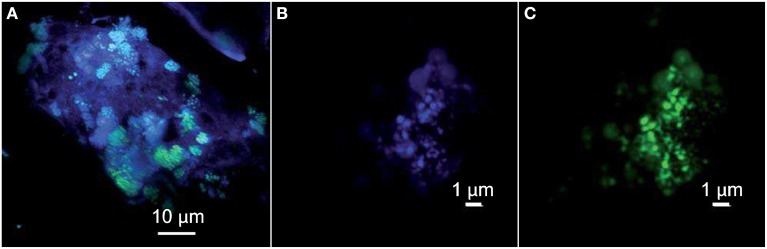
**Confocal Laser Scanning Microscopy stacked images obtained with a sequential excitation at 405 and 488 nm and fluorescence detection in the ranges 425–475 and 500–530 nm**. **(A)** Clusters of green Syto®9-stained cells interspersed in the chimney-forming minerals appearing as blue-violet. The archaeal cocci appear in turquoise thanks to the superimposition of their autofluorescence in blue and DNA staining with Syto®9 in green. **(B)** Archaeal cocci stained with DAPI (the self-fluorescence of F_420_ cofactor is also collected) and **(C)** hybridized with the FITC-labeled specific probe for *Euryarchaeota* EURY498.

Direct observations of carbonate samples by phase-contrast microscopy revealed dense microbial communities consisting mainly of thin long rods and cocci inhabiting pore spaces within the chimneys of ST09 (not shown). The cells seemed to be embedded in a matrix assumed to be microbial exopolysaccharides that could be responsible for the mucilaginous aspect around the central conduit of active chimneys (dark pink to brown colored, Figure 1E). Only the small cocci auto-fluoresced when excited at a wavelength of 420 nm, a characteristic of archaea containing cofactor 420 (F_420_) (Doddema and Vogels, 1978), a key coenzyme involved in methanogenesis (Thauer, 1998). These small fluorescent cocci occurred alone or typically in small spherical aggregates (of around 5 μm in diameter) of 10 to more than 20 tiny cells (around 0.5 μm in diameter) (Figure 4A). This organization resembles the structure known as cyst with a common outer wall surrounding coccoid cell aggregates, typical of the *Methanosarcina* genus and in particular of *M. acetivorans* (Sowers et al., 1984). The coupling of FISH to CSLM showed that the same aggregates of autofluorescent cocci hybridized with an *Euryarchaeota* specific probe, supporting the hypothesis that they belong to *Methanosarcinales* (Figures 4B,C).

LCMS were shown to be able to produce methane and to oxidize it in anaerobic conditions at the same time in single-species biofilms (Brazelton et al., 2012). The enhancement of both metabolisms by dihydrogen (H_2_) suggested syntrophic interactions between the two metabolically differentiated cell types (Brazelton et al., 2011). However, the cultivation of LCMS in pure culture has not been reported so far.

Many attempts were carried out to cultivate PHF *Methanosarcinales* by varying both the pH (from 8 to 10.5) and temperature conditions (30–60°C) and the electron donors (H_2_, acetate, formate, methanol, MMA, TMA). Limited methane production was detected after 5–12 days of incubation at 37°C during a few enrichment culture experiments using TMA or H_2_/CO_2_. Scarce tiny coccoid cells and thin filaments were observed in these mixed cultures, but the methanogenic populations could not be maintained in subcultures.

### Are there thermophilic archaea living in PHF?

**Figure 5:**
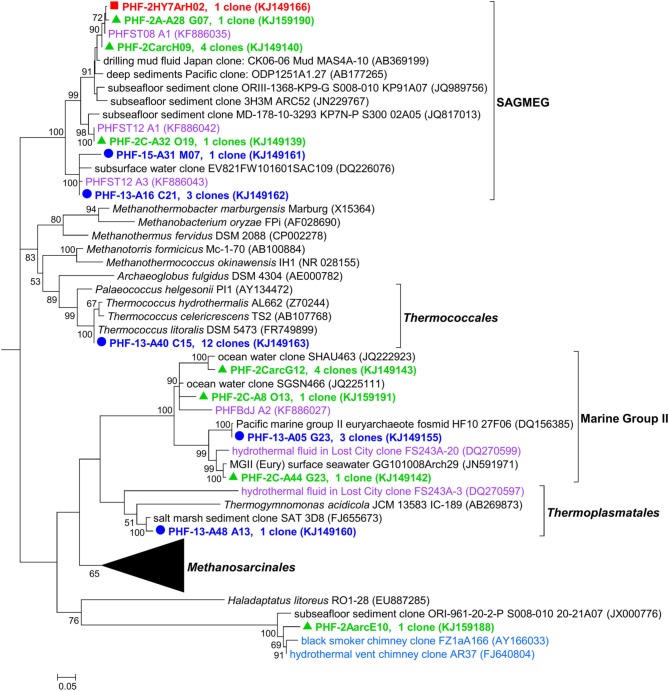
**Maximum likelihood phylogenetic tree representing the ***Euryarchaeota*** 16S rRNA gene phylotypes from ST09 chimneys**. Based on 489 positions in alignment. Bootstrap values < 50% are not shown. Clone libraries are distinguished by various symbols and colors: 2005 (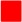 in red), 2010 (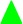 in green), and 2011 (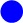 in blue). Purple font refers to phylotypes from marine hydrothermal serpentinized systems. Scale bar: number of substitutions per site.

Besides the unexpected discovery of LCMS in PHF, another surprising finding was the identification of many sequences closely related to cultivated members of the hyperthermophilic order *Thermococcales* and of environmental sequences related to hot biotopes, such as deep sea black smokers (Figure 5). Remarkably, the *Thermococcales* 16S rRNA sequences of Prony formed a single phylotype, representing 13% of the 2011 archaeal clone library, and identical to *Thermococcus litoralis* which was isolated from a shallow submarine volcanic spring in Italy (Neuner et al., 1990). *Thermococcales* are mostly heterotrophic, anaerobic, sulfur-reducers commonly found in marine hydrothermal vents. *A priori*, their growth conditions (optimum pH 6.5, up to pH 8.5, and a temperature range of 55–98°C) are not compatible with the physical and chemical conditions found in the PHF venting chimney (pH of 10.6 at ST09, and temperature at max 40°C). Scarce *Thermococcales* sequences were also reported in Lost City but only in fluid samples (Brazelton et al., 2006), which were almost identical to an isolate (related to *T. celer*) from a deep-sea volcano of the East Pacific Rise (Huber et al., 2006). Intriguingly, previous DGGE and clone library analyses from a cold subsurface oceanic sediment sample (2–3°C, 1.5 mbsf) collected at 3019 m water depth in the New Caledonian basin revealed low archaeal diversity dominated by *Thermococcales* clones that all grouped in a unique cluster (Roussel et al., 2009). Since no local hydrothermal activity was detected, the authors suggested that these *Thermococcales* could have rafted from PHF located about 200 km North East. The minimum temperature required for the growth of a *Thermococcus* is 40°C (Miroshnichenko et al., 2001), which questions the thermophilic nature of these archaea found in PHF. Are they an indicator of a hot subseafloor biotope linked to the exothermic reactions of the serpentinization process (Allen and Seyfried Jr, 2004; Takai et al., 2004) as suggested by Brazelton et al. (2006) for LCHF or does this PHF phyloptype represent a new moderately thermophilic ecotype of *T. litoralis*? At this stage we cannot conclude since our attempts to cultivate them at various temperatures (from 37 to 75°C) failed. This question also relates to the LCMS-like cells of PHF since LCMS were described to colonize only venting chimneys of LCHF, i.e., at temperatures in the hyperthermophilic range (between 55 and 95°C). The presence of LCMS-like cells in PHF at moderate temperatures (max 40°C) could therefore be the indication of the existence of a hot biotope beneath the PHF seafloor or may simply indicate that the PHF LCMS-like cells are, in fact, mesophilic archaea. Nonetheless, as previously mentioned, our preliminary attempts to cultivate these elusive LCMS were also not conclusive in the temperature range of 30–60°C. This addresses more generally the question of estimating the temperature lifestyle of uncultured prokaryotes in their natural environments. This problem was recently treated by Kimura et al. (2010, 2013) who estimated the growth temperatures of archaeal communities from the guanine-plus-cytosine contents (P_GC_) based on 16S rRNA sequences. The growth temperatures of the archaeal phylotypes found in PHF were predicted using their method (Table 3). For the sake of comparison, clones from similar environments, i.e., serpentinized systems or hydrothermal vents, and of a few representative archaeal strains were also reported. The predicted growth temperatures of the PHF *Thermococcales* clearly qualify them as hyperthermophilic with a temperature growth range very similar to that predicted and experimentally verified for their closest cultivated relative, *T. litoralis*. If these predictions hold true, all the PHF phylotypes assigned to the South African Gold Mine Euryarchaeotic Group SAGMEG (4% of the total archaea), could also be considered as thermophiles with an average predicted optimal growth temperature of about 70°C. This group is frequently associated with subsurface sediments and methane-rich environments (Parkes et al., 2014). As they were discovered in gold mine alkaline groundwater linked to a deep hot aquifer, they were assumed to be thermophiles (Takai et al., 2001) (Figure 5) although their actual morphology, metabolism and lifestyle remain unknown. Both *Thermococcales* and SAGMEG phylotypes could therefore indicate the existence of a hot subsurface biosphere in PHF.

**Table 3:** **Predicted growth temperature of uncultivated archaea and reference strains as a function of their 16S rRNA gene G+C percent (P_***GC***_) calculated from (Kimura et al., 2013)**.

**Clone**	**%G+C**	**T_*min*_**	**Topt**	**Tmax**	**Affiliation**
The Cedars ced_A01 (KC574884)	54.69	14	31	39	TCMS
**PHF_A45_G13 (50 clones)**	**54.94**	**15**	**32**	**40**	**TCMS**
**PHF_HY5ArA02 (80 clones)**	**54.63**	**14**	**30**	**39**	**TCMS**
**PHF_2A-A13-I03 (12 clones)**	**54.93**	**15**	**32**	**40**	**TCMS**
**PHF_2HY5ArE02 (15 clones)**	**52.25**	**4**	**19**	**27**	**LCMS**
**PHF_2AarcF09 (10 clones)**	**52.75**	**6**	**21**	**30**	**LCMS**
**13-A03_K23 (7 clones)**	**52.50**	**5**	**20**	**29**	**LCMS**
LC1022a1 (AY299516)	53.00	7	22	31	LCMS
LC1149a56 (AY299515)	53.25	8	24	32	LCMS
LC_AR37 (FJ640804)	51.88	2	17	25	LCMS
LC1231a51 (AY505046)	52.70	6	21	30	LCMS
*Methanosaeta_harundinacea*_6Ac (AY970347)	57.75	28 (25)	46 (37)	54 (45)	*Methanosarcinales*
*Methanothrix_thermophila*_(genome)	58.88	33	52 (55)	60	*Methanosarcinales*
**PHF_2CarcG12 (4 clones)**	**54.12**	**12**	**28**	**36**	**MG-II**
**PHF_13A05_G23 (3 clones)**	**57.08**	**25**	**43**	**51**	**MG-II**
LC_FS243A20_(DQ270599)	55.63	18	35	44	MG-II
**PHF_2CA44_G23 (1 clone)**	**54.75**	**15**	**31**	**40**	**MG-II**
**PHF_13A48_A13 (1 clone)**	**55.98**	**20**	**37**	**46**	***Thermoplasmatales***
**PHF_2AarcE10 (1clone)**	**52.38**	**4**	**19**	**28**	**Unknown deep-sea vent**
**PHF_A16_C21 (3 clones)**	**61.25**	**43**	**63**	**71**	**SAGMEG**
**PHF_2CA32_019 (1 clone)**	**61.62**	**45**	**65**	**73**	**SAGMEG**
**PHF_2CarcH09_(4 clones)**	**62.75**	**50**	**71**	**79**	**SAGMEG**
**PHF_2HY7ArH02 (1 clone)**	**62.24**	**47**	**68**	**76**	**SAGMEG**
3H3M_ARC52_(JN229767) (subseafloor_sediment)	62.25	47	68	76	SAGMEG
ODP1251A1.27_(AB177265) (deep_sediments,_Pacific)	61.38	44	64	72	SAGMEG
CK0606_Mud_MAS4A10_(AB369199) (drilling_mud_fluid,_Japan)	63.00	51	72	80	SAGMEG
**PHF_A40_C15 (12 clones)**	**65.75**	**63**	**86**	**93**	***Thermococcales***
*Thermococcus_litoralis* type strain: DSM5473 (FR749899)	65.87	63 (55)	86 (88)	94 (98)	*Thermococcales*

T_min_, T_opt_, and T_max_: minimal, optimal, and maximal predicted temperatures for growth, respectively (in bracket are values experimentally determined for such growth parameters of described species when available in the literature). Color shading code: blue for mesophilic (T_opt_ from 17 to 24° C); yellow for T_opt_ from 28 to 43°C; orange: T_opt_ from 46 to 72°C; red: T_opt_ > 80°C. Text in bold: PHF, clones from this study; LCMS, Lost City Methanosarcinales; MG-II, Marine Group II; SAGMEG, South African Gold Mine Euryarchaeotic Group; TCMS, The Cedars Methanosarcinales.

Using the same criterion, all the other archaea detected in PHF should be considered as mesophiles with a predicted optimal growth temperature (T_opt_) of 43°C at most. It notably includes all the *Methanosarcinales* (LCMS-like and TCMS-like) detected in PHF. Indeed, the average predicted growth temperatures of LCMS-like (T_opt_ 19°C, T_max_ 28°C) and those of TCMS-like (T_opt_ 31°C, T_max_ 40°C) were close to the temperatures recorded *in situ* at fluid vents. Based on this criterion, the predominance of *Methanosarcinales* sequences in all our chimney samples and our microscopic observations of typical *Methanosarcinales* morphotypes in these samples, we conclude that the *Methanosarcinales* found in PHF are mesophilic. What is puzzling is the fact that the predicted growth temperatures for true LCMS sequences, that we included in our analyses (from Brazelton et al., 2006) for comparison purposes, were also in the same range as those of LCMS-like sequences from PHF and even slightly lower than that of TCMS and TCMS-like. The hypothesis that LCMS sequences constitute an exception in Kimura's prediction method is unlikely since it was recently shown to apply to putatively thermophilic anaerobic methanotrophic archaea (ANME) from hot deep-sea vents (Merkel et al., 2013). Therefore, we may consider that the actual temperature growth range of these enigmatic archaea is much cooler than expected based on the fluid temperature measured at venting chimney outlets (70–90°C), and is probably more within the mesophilic range.

### Cosmopolitan *Thaum*- and *Eury-archaeota*

Archaea at PHF also includes phylotypes assigned to *Thaumarchaeota* and to *Euryarchaeota* Marine Group II which is not surprising because these two groups are the most abundant and frequently detected planktonic archaea (Delong, 2003; Galand et al., 2009) in various oceanic provinces (see the example of a tropical atoll, Michotey et al., 2012). *Thaumarchaeota* detected in PHF were particularly abundant in 2010 (70.6%) but less in 2011 (12.2%) clone libraries and showed high intra-phylum diversity with representatives of both the *Cenarchaeales* and the *Nitrososphaerales* orders (Supplementary Figure 4). The closest relatives of the PHF phylotypes were environmental sequences from hydrothermal vents, marine sediments and marine sponges, including clones of LCHF chimneys. The first isolated *Thaumarchaeota* members showed chemoautotrophic growth by aerobic oxidation of ammonia to nitrite and played an important role in the biogeochemical cycling of nitrogen (Schleper and Nicol, 2010). Members of the *Euryarchaeota* MG II were less abundant than *Thaumarchaeota* in PHF but like them, they were related to clones from either deep or surface oceanic waters, including one clone from LCHF. There is yet no cultivated *Euryarchaeota* MG II but a partial genome representative of this group was reconstructed from metagenomic data of surface seawater. As stated by Iverson et al. (2012) *sic* “The genome describes a motile, photo-heterotrophic cell focused on degradation of protein and lipids and clarifies the origin of proteorhodopsin.”

### *Firmicutes* dominate the PHF bacterial community

**Figure 6:**
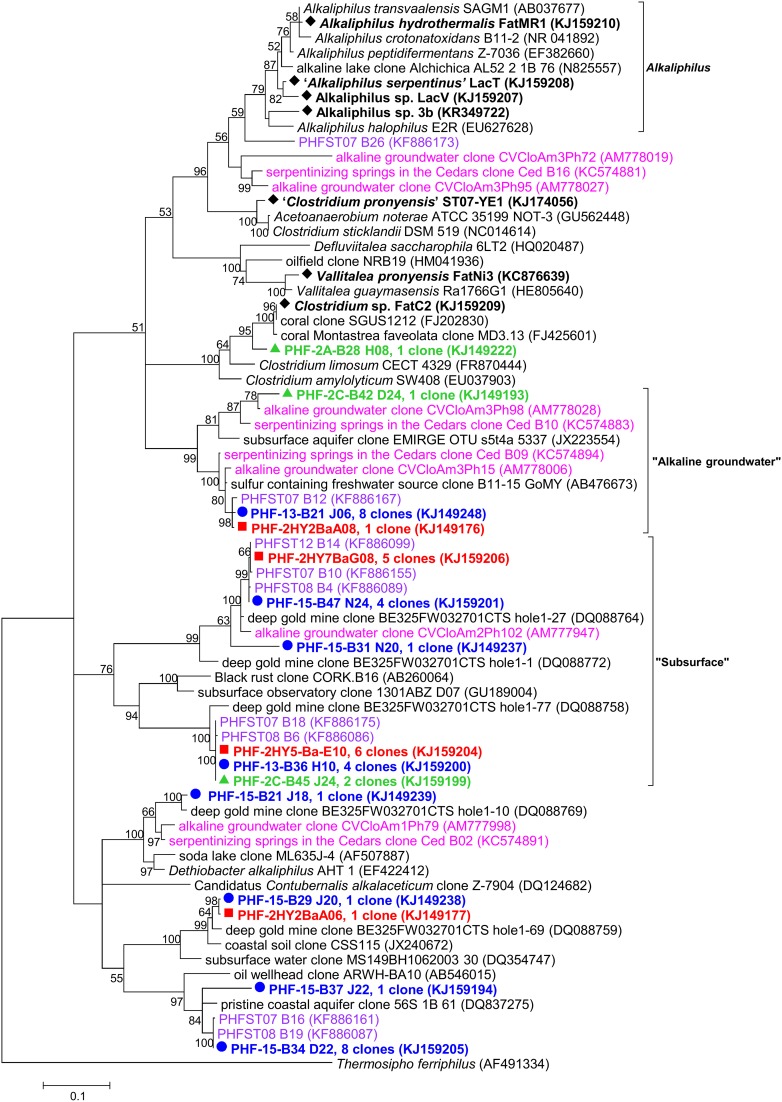
**Maximum likelihood phylogenetic tree of *Firmicutes* 16S rRNA gene phylotypes from ST09 chimneys**. Based on 563 positions in the alignment. Bootstrap values < 50% are not shown. Clone libraries are distinguished by various symbols and colors: 2005 (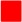 in red), 2010 (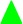 in green) and 2011 (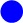 in blue). Isolated strains are indicated by ♦. Purple font refers to phylotypes from marine hydrothermal serpentinized systems, pink font refers to phylotypes from other terrestrial alkaline sites. Scale bar: number of substitutions per site.

*Firmicutes* was globally the most abundant bacterial phylum retrieved in this study although its proportion significantly varied between 2005, 2010, and 2011 (from 3.4 to 29.3%, Figures 2, 6). One particular cluster referred to here as “*subsurface”* was dominant and contained persistent phylotypes closely related to those found in a gold mine (Lin et al., 2006) and in CVA, Portugal (99.3% identity with CVCloAm2Ph102) (Tiago and Veríssimo, 2013). Another cluster of uncultivated organisms referred to here as “*alkaline groundwater”* encompasses two PHF phylotypes together with two OTUs of CVA and two OTU of The Cedars (Figure 6). The metabolism of these uncultivated members is so far unknown.

Assuming that the “true” indigenous fraction of the cultivable bacteria inhabiting the inside part of active PHF chimneys is suitably adapted to the *in situ* conditions, i.e., high pH fluids, low redox, and oxygen but high hydrogen and methane concentrations, we decided to apply anaerobic culture techniques to target these bacteria, guided with the results of the molecular survey. Only few enrichments cultures were successful. We tried to cultivate sulfate-reducing bacteria (SRB) from PHF chimney samples using various combinations of electron donors and acceptors at different pH and temperature conditions (see Materials and Methods). Unexpectedly, instead of SRB, we isolated from these cultures seven new strains of the order *Clostridiales*, all being strictly anaerobic fermentative heterotrophs. *Vallitalea pronyensis* sp. nov. was isolated from a sample of the ST09 chimney collected in 2010 (Ben Aissa et al., 2014). Since it is not alkaliphilic we assume that this bacterium develops in anaerobic niches of the chimney not directly in contact with pure hyperalkaline fluid. Four new strains of *Alkaliphilus* species were also isolated from PHF representing the first anaerobic alkaliphiles isolated from serpentinizing environments (Ben Aissa et al., 2015); they all are anaerobic and fermentative, able to grow by fermenting a variety of proteinaceaous compounds and sugars in moderately alkaline conditions (optimally at pH 8.7–9.5). Organic matter supporting their growth in the field may be metabolites produced by chemosynthetic primary producers (i.e., hydrogenotrophs, methanogens) or by their decays. Alternatively, they might be fed by small organic molecules (short chain hydrocarbons) abiotically generated by Fisher-Tropsch-type reactions linked to serpentinization (Proskurowski et al., 2008). Moreover, thermogenic organic compounds generated by the thermal degradation of deep serpentinite-hosted ecosystems (microbes or metazoans) can significantly contribute to the organic carbon balance in peridotite-hosted hydrothermal fields (Pasini et al., 2013).

**Figure 7:**
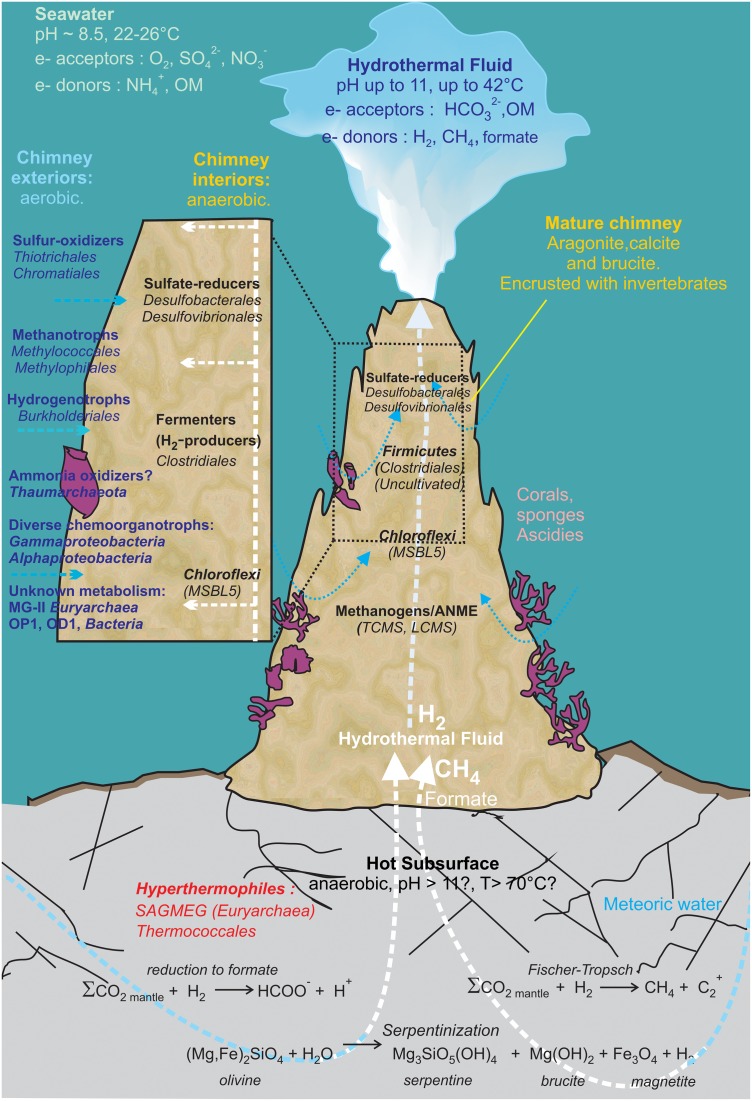
**Geomicrobiological model of the serpentinization-driven submarine ecosystem of PHF**. The physico-chemical conditions and mineralogy depicted in the zonation delimiting distinct microbial habitats are based on *in situ* measurements and lab analyses when available, or they have been hypothesized (such as in those in the deep subsurface settlement of the carbonate edifice). The potential electron donors and acceptors in the fluid and in seawater are given along with the putative metabolisms assigned to all the stable prokaryotes populations detected over 6-year period covered by this study. OM: Organic Matter. The scheme was inspired from Figure 7 of Brazelton et al. (2006).

*Clostridia* detected in various serpentinizing environments (Lost City, CVA, and the Cedars) were assumed to be abundant in the most extreme zones, i.e., the deepest anoxic parts, due to their high metabolic versatility and their autotrophy or mixotrophy capabilities (Figure 7) (Schrenk et al., 2013). Metagenomic data indicated that *Clostridiales* are major hydrogen producers in such alkaline ecosystems (Brazelton et al., 2012) which was further confirmed by culture-based approaches (Mei et al., 2014).

### Sulfur-cycling related bacteria

Sulfate-reducing bacteria of the class *Deltaproteobacteria* represented 5.3% from total bacterial clones (6% according to quantitative PCR). Despite their significant abundance in the original samples, culture media designed to target sulfate-reducers failed to enrich any *Deltaproteobacteria* (see above). *Desulfonatronum* (*Desulfovibrionales*), highlighted by 16S rRNA and *dsrB* sequences, appeared dominant and persistent in ST09 PHF chimneys over the 6-year period, suggesting its ecological significance in this ecosystem. Most of the *dsrB* OTUs were affiliated to *D. lacustre* (88.4–97.0% identity, representing 100, 97, and 54% of the bacterial clones respectively in the 2010, 2011, and 2005 libraries) while 16S phylotypes were related to *D. cooperativum* (Supplementary Figures 5, 6). *Desulfonatronum* species were isolated from various soda lakes and described as lithotrophic haloalkaliphilic sulfate-reducing bacteria, able to use hydrogen and a few organic compounds as electron donors (Zhilina et al., 2005). *Desulfobacterales* were also well represented by both 16S rRNA and *dsrB* sequences. In particular, *Desulfobulbaceae*-related *dsrB* OTUs were affiliated to the hydrogenotrophic *Desulfurivibrio alkaliphilus* (Sorokin et al., 2008) (91.4–93.6% identity, representing 29% and 3% of the 2005 and 2011 clone libraries). *Desulfobacteraceae*-related *dsrB* OTUs (PHF-23-D10 and OTU PHF-23-D21) were detected only in the 2005 sample and were related to halophilic sulfate-reducing bacteria, *Desulfotignum balticum* (95.7% identity) and *Desulfosarcina variabilis* (91.4% identity) respectively, which have often been detected in both pristine and contaminated marine environments (Joulian et al., 2001; Kuever et al., 2001; Gillan et al., 2005). *Desulfobacterales* are known to be associated with anaerobic methanotrophic archaea (ANME) (Schreiber et al., 2010); they represent putative syntrophic partners of anaerobic oxidation of methane (AOM) in association with ANME-3 related *Methanosarcinales*, as previously reported in deep sea hydrothermal vents and methane seeps (Knittel et al., 2003). At Lost City, *Desulfovibrionales* and *Desulfobacterales* were only rarely detected by 16S rDNA tag-sequencing and no corresponding *dsrB* gene was found (Brazelton et al., 2010a). Instead, *Desulfotomaculum* represented the dominant SRB detected in LCHF carbonate chimneys (Brazelton et al., 2006; Gerasimchuk et al., 2010) while they were not found in PHF ST09 chimneys. The differences observed in the patterns of SRB populations between LCHF and PHF might be explained by differences in fluid chemical composition (Supplementary Table 1) and in particular the salinity which is about 10 times higher in LCHF fluids than in PHF, whilst sulfate is about the same (1–4 mM in LCHF and ~3 mM in PHF). Thus *Desulfovibrionales* and *Desulfobacterales*, which dominate in PHF chimneys, may be better adapted to low salinity environments.

Additionally, within *Firmicutes, Dethiobacter*-like sequences identified in PHF clustered with clones from the terrestrial serpentinizing sites The Cedars and CVA (Figure 6). The type species *D. alkaliphilus* is an alkaliphilic and facultative hydrogenotrophic autotroph isolated from soda lake sediments (Sorokin et al., 2008).

Putative sulfur-oxidizing chemolithoautotrophs were also identified, related to the genera *Sulfurimonas (Epsilonproteobateria*, Supplementary Figure 7), *Sulfuritalea (Betaproteobateria*, Supplementary Figure 8) and mainly *Thioprofondum* and *Thiomicrospira* (*Gammaproteobacteria* Supplementary Figure 9). *Thiomicrospira* phylotypes identified in PHF were closely related to sequences from LCHF which were shown to be dominant in the fluids (Zhang et al., 2012). These bacteria most likely thrive in the peripheral part of the chimney walls, where seawater mixing with hydrothermal fluid provides optimum concentrations of electron donors, H_2_S, electron acceptor and O_2_ (this scenario is depicted in Figure 7). Putative (moderately) thermophilic bacteria were also evidenced in PHF chimneys, which belong to the genera *Thiomicrospira, Thioprofundum, Schlegelella, Deinococcus*-*Thermus* (see below). As discussed above for thermophilic archaea, these bacteria were most likely carried out by the fluid, ascending from a hot subsurface environment.

### *Chloroflexi* and candidate divisions

*Chloroflexi* was the second most abundant bacterial phylum in the three studied samples (representing up to one third of the bacterial sequences in the 2010 library) (Figure 2). The same trend was observed in the other underwater sites of PHF (in ST08 and ST12) we previously studied, where *Chloroflexi* represented up to 47% of total bacteria (Quéméneur et al., 2014). Four phylotypes found in ST09 chimneys were closely related to The Cedars Ced_B01 sequence (up to 97.1% identity), belonging to the candidate order named MSBL5 (Mediterranean Sea Brine Lake group 5) (Supplementary Figure 10) and three other phylotypes clustered with *Dehalococcoidia* sequences from marine subseafloor and siliciclastic sediments. Altogether, these new data support the hypothesis that *Chloroflexi* play a major role in the functioning of the PHF chimneys ecosystem, although the exact metabolisms of its representatives in such a natural environment are still largely unknown (Wasmund et al., 2013).

Candidate divisions OP1 and NPL-UPA2 were especially well represented (each 13.8% of total clones) (Supplementary Figure 11). Interestingly, a distinct clade within the candidate division NPL-UPA2 contained six PHF phylotypes (found each year) together with several LCHF and The Cedars OTUs, these sequences sharing up to 95.6% identity.

## Conclusion

This study provides a detailed description of the microbial communities from carbonate samples collected at an underwater site of the Prony hydrothermal field between 2005 and 2011. In PHF, the discharge of low-salinity, high-pH waters (of meteoric origin) produced by serpentinization of ultrabasic basement into a coastal marine environment forms large carbonate concretions and needles similar to those of LCHF. Our study shows that the microbial diversity found in PHF reflects this intermediate situation with populations characteristic of serpentinite environments similar to those found at on-land sites (i.e., *Chloroflexi* of the MSBL-5 cluster and TCMS from The Cedars, California) and to those characteristic of fully marine environments (i.e., LCMS). For comparison purpose, reference clones from PHF ST09 chimneys were compared with those from other serpentinizing environments (Supplementary Table 3).

These microorganisms may occupy diverse microhabitats inside the highly porous structure of hydrothermal carbonate concretions, where the mixing of local oxygenated seawater with the highly reduced CH_4_ and H_2_ rich hydrothermal fluid ascending from the subsurface produces large ranges and very sharp gradients of pH, redox potential (Eh), salinity and dissolved element concentrations (Figure 7). For example, *Methanosarcinales* and *Firmicutes* dominate in anoxic microhabitats irrigated by hydrothermal end member fluid (warm, reducing, and alkaline) as confirmed by cultivation experiments. Minority groups representing (hyper)thermophilic lineages such as *Thermococcales* and SAGMEG in archaea or *Thermus* in bacteria were also consistently detected in the PHF chimneys but their predicted growth temperatures strongly suggest they were carried out by a fluid ascending from a hot subsurface environment heated by serpentinization reactions. Representatives of other metabolic groups are distributed along the physicochemical gradients depending mainly on their electron acceptor requirements and their tolerance to oxygen. Typically, SRB (*Deltaproteobacteria*), although most of them are oxygen tolerant (Muyzer and Stams, 2008), occupy anaerobic microhabitats where sulfate is provided by the mixing of the sulfate-free hydrothermal fluid with seawater. At the oxic pole of the gradient, microaerophilic or aerobic microorganisms such as methylotrophic bacteria (*Methylococcales, Methylophilales*), hydrogen-oxidizing (*Burkholderiales*) and sulfur-oxidizing bacteria (*Chromatiales, Thiotrichales*) thrives by oxidation of H_2_, CH_4_, and H_2_S. Other marine aerobic bacteria and archaea found in PHF and LCHF do not seem to be specific of serpentinite environments as they belong to cosmopolitan marine groups such as *Thaumarchaeota* or MG-II. Altogether, these microorganisms certainly play important roles in budgets of methane, hydrogen, and sulfur in PHF as depicted in Figure 7. However, these scenarios remain largely hypothetical since most of the microorganisms thriving in serpentinizing environments have not as yet been able to be cultivated. Yet, the first anaerobic alkaliphilic bacteria from a serpentinized ecosystem could be isolated from PHF ST09 chimneys. They belong to *Firmicutes*, a dominant phylum in PHF and in LCHF, generally known for their versatile metabolic capabilities but whose exact roles in the chimney ecosystem remain to be clarified.

### Conflict of interest statement

The authors declare that the research was conducted in the absence of any commercial or financial relationships that could be construed as a potential conflict of interest.
