# Supplementary material for: Microbial diversity in a submarine carbonate edifice from the serpentinizing hydrothermal system of the Prony Bay (New Caledonia) over a 6-year period
Source: Front Microbiol. 2015 Aug 27;6:857. doi: 10.3389/fmicb.2015.00857 (PMC4551099; doi:10.3389/fmicb.2015.00857)
Supplement: Supplementary file 1 [file Table1.PDF]

Supplementary Material

Microbial diversity in a submarine hydrothermal chimney from the serpentinized system of the Prony Bay (New Caledonia) over a 6 years period.

Anne Postec1*, Marianne Quéméneur1, Méline Bes1, Nan Mei1, Fatma Benaïssa1, Claude Payri2, Bernard Pelletier2, Christophe Monnin3, Linda Dombrowsky1,2, Bernard Ollivier1, Emmanuelle Gérard5, Céline Pisapia5, Martine Gérard4, Bénédicte Ménez5, Gaël Erauso1*.

1 Aix Marseille Université, CNRS/INSU, IRD, Mediterranean Institute of Oceanography, UM110, 13288 Marseille, France

2 Institut pour la Recherche et le Développement, Centre de Nouméa, promenade Laroque, 98848 Nouméa, Nouvelle-Calédonie

3 Géosciences Environnement Toulouse, UMR 5563, 14 avenue Édouard Belin, 31400 Toulouse

4 Institut de Minéralogie et de Physique des Milieux Condensés, 4 place Jussieu, 75005 Paris, France

5 Institut de Physique du Globe de Paris, Sorbonne Paris Cité, Univ. Paris Diderot, CNRS, 75005 Paris, France

*** Correspondence: anne.postec@~~univ-amu.fr~~ and gael.erauso@~~univ-amu.fr~~**

Supplementary Table 1. Geochemical properties of marine and terrestrial serpentinizing sites (modified from Suzuki et al., 2013).

	Prony (New Caledonia)	Lost City (Mid Atlantic Ridge)	Cabeço de Vide (Portugal)	The Cedars (USA)		
	**ST09**		**AC3**	**BS1**	**BS5**	**GPS1**
**pH**	10.6	9-9.8	11.4	11.5	11.6	11.9
**N_2 (% by vol)**	67-69*	-	-	49.6	53.6	36.6
**H_2 (% by vol)**	19-24*	-	-	39.2	34	50.9
**CH_4 (% by vol)**	6-13*	-	-	6.5	5.3	7.4
**N_2 (aq) mM**	-	-	-	-	-	-
**H_2 (aq) mM**	-	0.25-0.43	-	-	-	-
**CH_4 (aq) mM**	-	0.13-0.28	-	-	-	-
**Na^+ (mM)**	6.46	479-485	2.26	2.0	1.98	14.69
**K^+ (mM)**	0.98	-	0.12	0.038	0.03	0.13
**Ca^{2+} (mM)**	2.76	21.0-23.3	0.58	-	1.17	0.94
**Mg^{2+} (mM)**	2.768	9-19	<dl	-	0.036	0.004
**PO_4^{3-} (mM)**	-	-	-	<dl	<dl	<dl
**HCO^{3-} (mM)**	-	-	-	<dl	<dl	<dl
**Cl^- (mM)**	50.58	546-549	1.48	1.7	1.49	8.73
**NO_3^- (mM)**	-	-	<dl	<dl	<dl	<dl
**SO_4^{2-} (mM)**	1.72	5.9-12.9	0.03	<dl	0.001	<dl

* from Bain des Japonais and Rivière des Kaoris sites (Monnin et al. 2014). - ; not available, <dl ; below detection limit.
