# Supplementary material for: Microbial diversity in a submarine carbonate edifice from the serpentinizing hydrothermal system of the Prony Bay (New Caledonia) over a 6-year period
Source: Front Microbiol. 2015 Aug 27;6:857. doi: 10.3389/fmicb.2015.00857 (PMC4551099; doi:10.3389/fmicb.2015.00857)
Supplement: Supplementary file 2 [file Table2.PDF]

Supplementary Material

Microbial diversity in a submarine hydrothermal chimney from the serpentinized system of the Prony Bay (New Caledonia) over a 6 years period.

Anne Postec1*, Marianne Quéméneur1, Méline Bes1, Nan Mei1, Fatma Benaïssa1, Claude Payri2, Bernard Pelletier2, Christophe Monnin3, Linda Dombrowsky1,2, Bernard Ollivier1, Emmanuelle Gérard5, Céline Pisapia5, Martine Gérard4, Bénédicte Ménez5, Gaël Erauso1*.

1 Aix Marseille Université, CNRS/INSU, IRD, Mediterranean Institute of Oceanography, UM110, 13288 Marseille, France

2 Institut pour la Recherche et le Développement, Centre de Nouméa, promenade Laroque, 98848 Nouméa, Nouvelle-Calédonie

3 Géosciences Environnement Toulouse, UMR 5563, 14 avenue Édouard Belin, 31400 Toulouse

4 Institut de Minéralogie et de Physique des Milieux Condensés, 4 place Jussieu, 75005 Paris, France

5 Institut de Physique du Globe de Paris, Sorbonne Paris Cité, Univ. Paris Diderot, CNRS, 75005 Paris, France

*** Correspondence: anne.postec@~~univ-amu.fr~~ and gael.erauso@~~univ-amu.fr~~**

Supplementary Table 2. Primers used in this study for detection of 16S rRNA genes and functional genes (*dsrAB* and *mcrA* genes).

Primer	Sequence (5'→3')	Reference
**Bacterial 16S rRNA genes**		
**27F**	AGA GTT TGA TCM TGG CTC AG	(Lane, 1991)
**907R**	CCG TCA ATT CMT TTR AGT TT	(Lane, 1991)
**341F***	CCT ACG GGA GGC AGC AG	(Muyzer et al., 1993)
**518R**	ATT ACC GCG GCT GCT GG	(Muyzer et al., 1993)
**Archaeal 16S rRNA genes**		
**109F**	ACK GCT CAG TAA CAC GT	(Webster et al., 2006)
**958R**	YCC GGC GTT GAM TCC AAT T	(Webster et al., 2006)
**344F***	ACG GGG HGC AGC AGG CGC GA	(Raskin et al., 1994)
**519R**	GWA TTA CCG CGG CKG CTG	(Amann et al., 1995)
**EURY498**	CTT GCC CRG CCC TT	(Loy et al., 2003)
***mcrA* genes**		
**MLF**	GGT GGT GTM GGA TTC ACA CAR TAY GCW ACA GC	(Luton et al., 2002)
**MLR**	TTC ATT GCR TAG TTW GGR TAG TT	(Luton et al., 2002)
**ME2R’**	TCA TBG CRT AGT TDG GRT AGT	(Nunoura et al., 2008)
**ME3MF**	ATG TCN GGT GGH GTM GGS TTY AC	(Nunoura et al., 2008)
**ME3MF-e**	ATG AGC GGT GGT GTC GGT TTC AC	(Nunoura et al., 2008)
***dsrAB* genes**		
**DSRp2060F**	CAA CAT CGT YCA YAC CCA GGG	(Geets et al., 2006)
**DSR4R**	GTG TAG CAG TTA CCG CA	(Wagner et al., 1998)

* A 40-bp GC clamp was added to the 5’ end for DGGE analysis: (5’-CGC CCG CCG CGC GCG GCG GGC GGG GCG GGG GCA CGG GGG G-3’) (Sheffield et al., 1989).

References

Amann, R.I., Ludwig, W., and Schleifer, K.-H. (1995). Phylogenetic identification and *in situ* detection of individual microbial cells without cultivation. *Microbiol. Rev.* 59**,** 143-169.

Geets, J., Borremans, B., Diels, L., Springael, D., Vangronsveld, J., Van Der Lelie, D., and Vanbroekhoven, K. (2006). DsrB gene-based DGGE for community and diversity surveys of sulfate-reducing bacteria. *J. Microbiol. Meth.* 66**,** 194-205. doi: 10.1016/j.mimet.2005.11.002.

Lane, D.J. (1991). "16S/23S rRNA sequencing.," in *Nucleic Acid Tech. Bact. Syst.*, ed. E.S.M. Goodfellow. (John Wiley and Sons, New York), 115-175.

Loy, A., Horn, M., and Wagner, M. (2003). probeBase: an online resource for rRNA-targeted oligonucleotide probes. *Nucleic Acids Res.* 31**,** 514-516. doi: 10.1093/nar/gkg016.

Luton, P.E., Wayne, J.M., Sharp, R.J., and Riley, P.W. (2002). The *mcrA* gene as an alternative to 16S rRNA in the phylogenetic analysis of methanogen populations in landfill. *Microbiology* 148**,** 3521-3530.

Nunoura, T., Oida, H., Miyazaki, J., Miyashita, A., Imachi, H., and Takai, K. (2008). Quantification of *mcrA* by fluorescent PCR in methanogenic and methanotrophic microbial communities. *FEMS Microbiol. Ecol.* 64**,** 240-247.

Raskin, L., Poulsen, L.K., Noguera, D.R., Rittmann, B.E., and Stahl, D.A. (1994). Quantification of methanogenic groups in anaerobic biological reactors by oligonucleotide probe hybridization. *Appl. Environ. Microbiol.* 60**,** 1241-1248.

Sheffield, V.C., Cox, D.R., Lerman, L.S., and Myers, R.M. (1989). Attachment of a 40-base pair G+C-rich sequence (GC- clamp) to genomic DNA fragments by the polymerase chain reaction results in improved detection of single-base changes. *Proc. Natl. Acad. Sci. U.S.A.* 86, 232-236.

Wagner, M., Roger, A.J., Flax, J.L., Brusseau, G.A., and Stahl, D.A. (1998). Phylogeny of dissimilatory sulfite reductases supports an early origin of sulfate respiration. *J. Bacteriol.* 180**,** 2975-2982.

Webster, G., John Parkes, R., Cragg, B.A., Newberry, C.J., Weightman, A.J., and Fry, J.C. (2006). Prokaryotic community composition and biogeochemical processes in deep subseafloor sediments from the Peru Margin. *FEMS Microbiol. Ecol.* 58**,** 65-85. doi: 10.1111/j.1574-6941.2006.00147.x.
