# Supplementary material for: Microbial diversity in a submarine carbonate edifice from the serpentinizing hydrothermal system of the Prony Bay (New Caledonia) over a 6-year period
Source: Front Microbiol. 2015 Aug 27;6:857. doi: 10.3389/fmicb.2015.00857 (PMC4551099; doi:10.3389/fmicb.2015.00857)
Supplement: Supplementary file 3 [file Table3.PDF]

frontiers

Supplementary Material

Microbial diversity in a submarine hydrothermal chimney from the serpentinized system of the Prony Bay (New Caledonia) over a 6 years period.

Anne Postec1*, Marianne Quéméneur1, Méline Bes1, Nan Mei1, Fatma Benaïssa1, Claude Payri2, Bernard Pelletier2, Christophe Monnin3, Linda Dombrowsky1,2, Bernard Ollivier1, Emmanuelle Gérard5, Céline Pisapia5, Martine Gérard4, Bénédicte Ménez5, Gaël Erauso1*.

1 Aix Marseille Université, CNRS/INSU, IRD, Mediterranean Institute of Oceanography, UM110, 13288 Marseille, France

2 Institut pour la Recherche et le Développement, Centre de Nouméa, promenade Laroque, 98848 Nouméa, Nouvelle-Calédonie

3 Géosciences Environnement Toulouse, UMR 5563, 14 avenue Édouard Belin, 31400 Toulouse

4 Institut de Minéralogie et de Physique des Milieux Condensés, 4 place Jussieu, 75005 Paris, France

5 Institut de Physique du Globe de Paris, Sorbonne Paris Cité, Univ. Paris Diderot, CNRS, 75005 Paris, France

*** Correspondence: anne.postec@~~univ-amu.fr~~ and gael.erauso@~~univ-amu.fr~~**

Supplementary Table 3. Comparison of the PHF microbial community with other serpentinizing environments*.

OTU reference clone**	OTU size	% sequences / year			Taxonomy	Blast hit	% Identity	% Coverage	Alignement length	Source
		2005	2010	2011						
PHF-HY5ArA02 (KJ149164)	80	83.3				clone_Ced_A01 (KC574884)	98.75	100	796	
PHF-13-A45-G13 (KJ149156)	50			54.9	*Methanosarcinales*	clone_Ced_A01 (KC574884)	99.5	100	799	The Cedars
PHF-2A-A13-I03 (KJ149144)	12		10.1			clone_Ced_A01 (KC574884)	98.5	100	799	
PHF-2HY5ArE02 (KJ149165)	15	15.6				clone_SGXU588 (FJ791633)	95.88	100	796	
PHF-2AarcF09 (KJ149145)	10		8.4			clone_SGXU588 (FJ791633)	96.5	100	800	
PHF-13-A03-K23 (KJ149159)	7			7.7	*Methanosarcinales*	clone_SGXU457 (FJ791612)	95.51	100	800	LCHF
PHF-15-A33-A09 (KJ149157)	1			1.1		clone_SGXU755 (FJ791575)	96.62	100	800	
PHF-13-A49-I13 (KJ149158)	1			1.1		clone_SGXU541 (FJ791597)	95.88	100	800	
PHF-2AarcE07 (KJ149148)	29		24.4			clone_LC1231a82 (AY505052)	97.12	100	799	
PHF-13-A10-O21 (KJ149154)	4			4.4		clone_LC1231a82 (AY505052)	97.62	100	799	
PHF-2AarcE06 (KJ149146)	3		2.5			clone_LC1231a82 (AY505052)	96.7	100	799	
PHF-2AarcE03 (KJ149147)	3		2.5			clone_LC1231a82 (AY505052)	99.0	100	799	
PHF-13-A02-M23 (KJ149153)	1			1.1	*Thaumarchaeota*	clone_LC1231a82 (AY505052)	95.2	100	799	LCHF
PHF-2A-A40-O09 (KJ149135)	5		4.2			clone_LC1231a80 (AY505051)	96.1	100	799	
PHF-15-A26-C07 (KJ149150)	2			2.2		clone_LC1231a80 (AY505051)	95.1	100	799	
PHF-2C-A39-M21 (KJ149141)	1		0.8			clone_LC1231a80 (AY505051)	98.5	100	799	
PHF-15-A23-M05 (KJ149151)	3			3.3		clone_LC1231a51 (AY505046)	98.6	100	799	
PHF-2CarcH11 (KJ149133)	6		5.0		*Thaumarchaeota*	clone_F160cmFL245 (JN002684)	95.9	100	797	Leka ophiolite complex
PHF-2AarcE02 (KJ149137)	3		2.5		*Thaumarchaeota*	clone_DSA_OTU_2 (EF414498)	96.4	100	799	Mariana forearc mud volcanos
PHF-15-A24-O05 (KJ149152)	1			1.1		clone_DSA_OTU_1 (EF414497)	96.7	100	798	
PHF-13-A40-C15 (KJ149163)	12			13.2	*Thermococcales*	*Thermococcus* sp.Tc-S-85 (AB095157)	98.5	100	802	Central Indian Ridge
PHF-2C-B47-N24 (KJ149191)	15		12.6			clone_Ced_B01 (KC574890)	97.1	100	854	
PHF-13-B5-J02 (KJ149246)	5			5.4	*Chloroflexi*	clone_Ced_B01 (KC574890)	97.5	100	855	The Cedars
PHF-2A-B10-D04 (KJ149230)	3		2.5			clone_Ced_B01 (KC574890)	96.8	100	787	
PHF-HY7BaG04 (KJ149167)	2	2.8				clone_Ced_B01 (KC574890)	96.7	100	787	

PHF-2A-B16-P04 (KJ149228)	3		2.5			clone_NS1B1_K15 (KC574967)	97.2	76.6	849	
PHF-2HY3BaC10 (KJ149172)	9	12.7			*Betaproteobacteria*	clone_LC1524B-50 (DQ270636)	99.6	98.9	878	LCHF
PHF-13-B7-N02 (KJ149245)	1			1.1	*Betaproteobacteria*	clone_F160cmL260 (JN002878)	95.1	78.3	886	Leka ophiolite complex
PHF-2CbacC03 (KJ149184)	1		0.8		*Betaproteobacteria*	clone_lagoon3_O12 (KC574828)	99.2	92.5	798	The Cedars
PHF-13-B21-J06 (KJ149248)	8			8.7	*Firmicutes*	clone_CVCloAm3Ph15 (AM778006)	97.4	90.4	908	CVA
PHF-2HY7-BaG08 (KJ159206)	5	7.0				clone_CVCloAm2Ph102 (AM777947)	97.7	100	817	
PHF-15-B47-N24 (KJ159201)	4			4.3		clone_CVCloAm2Ph102 (AM777947)	98.1	100	827	
PHF-2HY2BaA08 (KJ149176)	1	1.4				clone_CVCloAm3Ph15 (AM778006)	97.0	90.4	908	
PHF-15-B21-J18 (KJ149239)	1			1.1		clone_CVCloAm2Ph23 (AM777965)	97.6	100	782	
PHF-2C-B42-D24 (KJ149193)	1		0.8			clone_CVCloAm3Ph98 (AM778028)	95.5	100	863	
PHF-13-B3-F02 (KJ149247)	4			4.3	*Alphaproteobacteria*	clone_F155cmContig9 (JN002777)	95.2	96	833	Leka ophiolite complex
PHF-2C-B2-D14 (KJ149202)	1		0.8			clone_F155cmContig9 (JN002777)	95.2	96	833	
PHF-15-B16-P16 (KJ149243)	2			2.2	*Alphaproteobacteria*	clone_SGXT605 (FJ792016)	95.9	100	820	LCHF
PHF-2A-B9-B04 (KJ149213)	1		0.8			clone_SGXT398 (FJ79183)	96.0	100	823	
PHF-15-B28-H20 (KJ159192)	1			1.1	*Gammaproteobacteria*	clone_SGYF714 (FJ792439)	97.5	84.1	811	LCHF
PHF-2AbacB08 (KJ149210)	1		0.8			clone_SGXT449 (FJ791881)	96.1	100	876	
PHF-2A-B48-P12 (KJ149216)	1		0.8			clone_SGXT626 (FJ792035)	95.1	100	892	
PHF-13-B11-F04 (KJ149254)	2			2.2	*Deinococcus-Thermus*	clone_CVCloAm1Ph47 (AM777992)	98.7	100	776	CVA

* Results of blastn searches : only hits > 95% identity with sequences from serpentinizing environments deposited in Genbank (accession number in the brakets) are shown. References for LCHF (Mid Atlantic Ridge): Schrenk et al., 2004; Brazelton et al., 2006; Brazelton et al., 2010; Gerasimchuk et al., 2010; Roussel et al., 2011; for The Cedars: Suzuki et al., 2013; for CVA: Tiago et al., 2004, Tiago and Veríssimo, 2013; for Kairei (Central Indian Ridge) : Takai et al., 2004; for Mariana Forearc: Curtis et al., 2012; for Rainbow (Mid Atlantic Ridge): Roussel et al., 2011; for Leka (Norway): Daae et al., 2013; for Del Puerto Ophiolite (California): Blank et al., 2009

** In red, sequences from 2005 clone library, in green from 2010 and in blue from 2011.

Blank, J., Green, S., Blake, D., Valley, J., Kita, N., Treiman, A., and Dobson, P. (2009). An alkaline spring system within the Del Puerto Ophiolite (California, USA): a Mars analog site. *Planet. Sp. Sci.* 57, 533-540.

Brazelton, W.J., Schrenk, M.O., Kelley, D.S., and Baross, J.A. (2006). Methane- and sulfur-metabolizing microbial communities dominate the Lost City Hydrothermal Field Ecosystem. *Appl. Environ. Microb.* 72, 6257-6270. doi: 10.1128/aem.00574-06.

Brazelton, W.J., Sogin, M.L., and Baross, J.A. (2010). Multiple scales of diversification within natural populations of archaea in hydrothermal chimney biofilms. *Environ. Microbiol. Rep.* 2, 236-242.

Curtis, A.C., Wheat, C.G., Fryer, P., and Moyer, C.L. (2012). Mariana Forearc serpentinite mud volcanoes harbor novel communities of extremophilic Archaea. *Geomicrobiol. J.* 30, 430-441. doi: 10.1080/01490451.2012.705226.

Daae, F.L., Økland, I., Dahle, H., Jørgensen, S.L., Thorseth, I.H., and Pedersen, R.B. (2013). Microbial life associated with low-temperature alteration of ultramafic rocks in the Leka ophiolite complex. *Geobiology* 11, 318-339.

Gerasimchuk, A., Shatalov, A., Novikov, A., Butorova, O., Pimenov, N., Lein, A., Yanenko, A., and Karnachuk, O. (2010). The search for sulfate-reducing bacteria in mat samples from the lost city hydrothermal field by molecular cloning. *Microbiology* 79, 96-105.

Roussel, E.G., Konn, C., Charlou, J.-L., Donval, J.-P., Fouquet, Y., Querellou, J., Prieur, D., and Cambon Bonavita, M.-A. (2011). Comparison of microbial communities associated with three Atlantic ultramafic hydrothermal systems. *FEMS Microb. Ecol.* 77, 647-665.

Schrenk, M.O., Kelley, D.S., Bolton, S.A., and Baross, J.A. (2004). Low archaeal diversity linked to subseafloor geochemical processes at the Lost City Hydrothermal Field, Mid-Atlantic Ridge. *Environ. Microbiol.* 6, 1086-1095.

Suzuki, S., Ishii, S.I., Wu, A., Cheung, A., Tenney, A., Wanger, G., Kuenen, J.G., and Nealson, K.H. (2013). Microbial diversity in The Cedars, an ultrabasic, ultrareducing, and low salinity serpentinizing ecosystem. *Proc. Natl. Acad. Sci. U.S.A.* doi: 10.1073/pnas.1302426110.

Takai, K.H., Gamo, T., Tsunogai, U., Nakayama, N., Hirayama, H., Nealson, K.H., and Horikoshi, K. (2004). Geochemical and microbiological evidence for a hydrogen-based, hyperthermophilic subsurface lithoautotrophic microbial ecosystem (HyperSLiME) beneath an active deep-sea hydrothermal field. *Extremophiles* 8, 269 - 282.

Tiago, I., Chung, A.P., and Verissimo, A. (2004). Bacterial diversity in a nonsaline alkaline environment: heterotrophic aerobic populations. *Appl. Environ. Microb.* 70, 7378-7387. doi: 10.1128/aem.70.12.7378-7387.2004.

Tiago, I., and Veríssimo, A. (2013). Microbial and functional diversity of a subterrestrial high pH groundwater associated to serpentinization. *Environ. Microbiol.* 15, 1687-1706.
