# Supplementary material for: Microbial diversity in a submarine carbonate edifice from the serpentinizing hydrothermal system of the Prony Bay (New Caledonia) over a 6-year period
Source: Front Microbiol. 2015 Aug 27;6:857. doi: 10.3389/fmicb.2015.00857 (PMC4551099; doi:10.3389/fmicb.2015.00857)
Supplement: Supplementary file 6 [file Image3.PDF]

Supplementary Material

Microbial diversity in a submarine hydrothermal chimney from the serpentinized system of the Prony Bay (New Caledonia) over a 6 years period.

Anne Postec1*, Marianne Quéméneur1, Méline Bes1, Nan Mei1, Fatma Benaïssa1, Claude Payri2, Bernard Pelletier2, Christophe Monnin3, Linda Dombrowsky1,2, Bernard Ollivier1, Emmanuelle Gérard5, Céline Pisapia5, Martine Gérard4, Bénédicte Ménez5, Gaël Erauso1*.

1 Aix Marseille Université, CNRS/INSU, IRD, Mediterranean Institute of Oceanography, UM110, 13288 Marseille, France

2 Institut pour la Recherche et le Développement, Centre de Nouméa, promenade Laroque, 98848 Nouméa, Nouvelle-Calédonie

3 Géosciences Environnement Toulouse, UMR 5563, 14 avenue Édouard Belin, 31400 Toulouse

4 Institut de Minéralogie et de Physique des Milieux Condensés, 4 place Jussieu, 75005 Paris, France

5 Institut de Physique du Globe de Paris, Sorbonne Paris Cité, Univ. Paris Diderot, CNRS, 75005 Paris, France

*** Correspondence: anne.postec@~~univ-amu.fr~~ and gael.erauso@u~~niv-amu.fr~~**

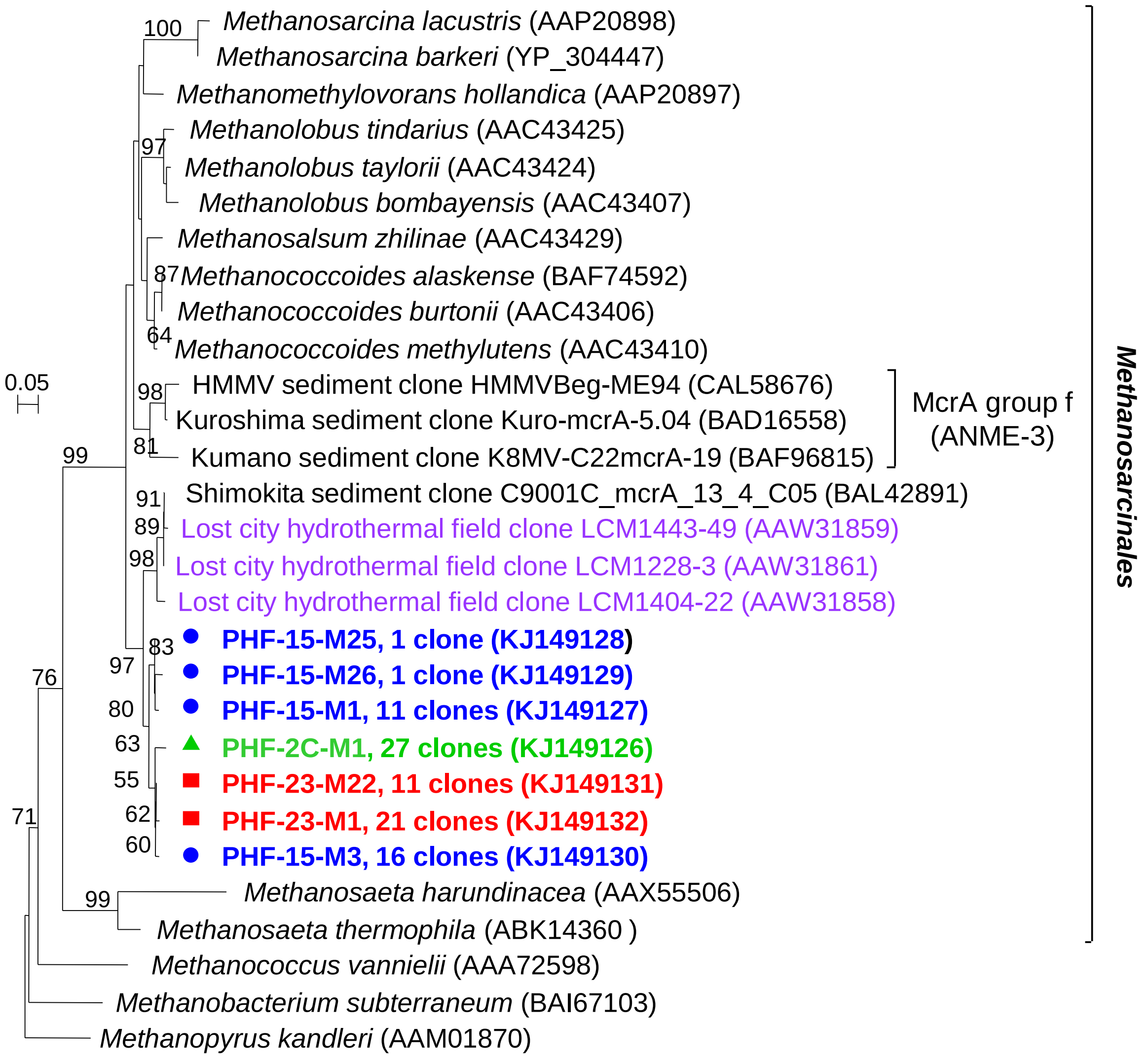

Supplementary Figure 3. Phylogenetic tree representing *mcrA* translated gene sequences. The tree was constructed using the neighbor joining method. Bootstrap values <70% are not shown. Clone libraries are distinguished by various ~~markers~~ and colors: 2005 (■ in red), 2010 (▲ in green) and 2011 (● in blue). Purple font ~~denotes~~ phylotypes from marine serpentinized settings. After each OTU name is indicated the number of clones retrieved for this OTU. Scale bar, number of substitution per site.
