# Supplementary material for: Microbial diversity in a submarine carbonate edifice from the serpentinizing hydrothermal system of the Prony Bay (New Caledonia) over a 6-year period
Source: Front Microbiol. 2015 Aug 27;6:857. doi: 10.3389/fmicb.2015.00857 (PMC4551099; doi:10.3389/fmicb.2015.00857)
Supplement: Supplementary file 9 [file Image6.PDF]

Supplementary Material

Microbial diversity in a submarine hydrothermal chimney from the serpentinized system of the Prony Bay (New Caledonia) over a 6 years period.

Anne Postec[1]*, Marianne Quéméneur[1], Méline Bes[1], Nan Mei[1], Fatma Benaïssa[1], Claude Payri[2], Bernard Pelletier[2], Christophe Monnin[3], Linda Dombrowsky[1,2], Bernard Ollivier[1], Emmanuelle Gérard[5], Céline Pisapia[5], Martine Gérard[4], Bénédicte Ménez[5], Gaël Erauso[1]*.

1 Aix Marseille Université, CNRS/INSU, IRD, Mediterranean Institute of Oceanography, UM110, 13288 Marseille, France

2 Institut pour la Recherche et le Développement, Centre de Nouméa, promenade Laroque, 98848 Nouméa, Nouvelle-Calédonie

3 Géosciences Environnement Toulouse, UMR 5563, 14 avenue Édouard Belin, 31400 Toulouse

4 Institut de Minéralogie et de Physique des Milieux Condensés, 4 place Jussieu, 75005 Paris, France

5 Institut de Physique du Globe de Paris, Sorbonne Paris Cité, Univ. Paris Diderot, CNRS, 75005 Paris, France

*** Correspondence: anne.postec@~~univ-amu.fr~~ and gael.erauso@~~univ-amu.fr~~**

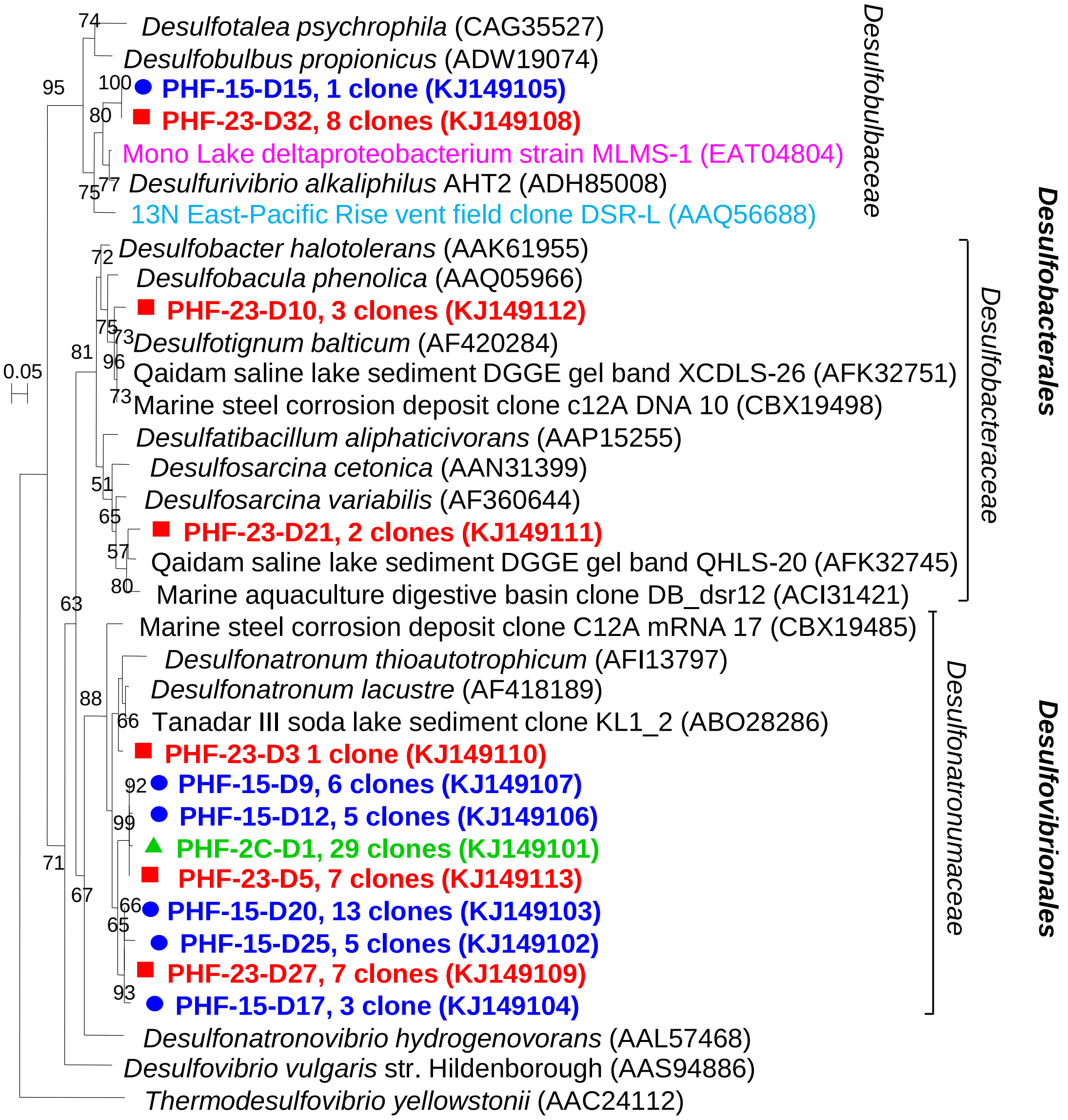

Supplementary Figure 6. Phylogenetic tree representing *dsrB* translated gene sequences. The tree was constructed using the neighbor joining method. Bootstrap values <50% are not shown. Clone libraries are distinguished by various ~~markers~~ and colors: 2005 (■ in red), 2010 (▲ in green) and 2011 (● in blue). Pink font ~~denotes~~ phylotypes from other terrestrial alkaline sites, light blue ~~denotes~~ phylotypes from hydrothermal. After each OTU name is indicated the number of clones ~~retrieved~~ for this OTU. Scale bar, number of substitution per site.
